# Diversification across the Australian Monsoonal Tropics: Comparing phylogeographic and demographic patterns within and between species of *Cryptoblepharus* skinks

**DOI:** 10.1038/s41437-026-00843-8

**Published:** 2026-04-18

**Authors:** Sofía I. Hayden Bofill, Sally Potter, Ana C. Afonso Silva, Craig Moritz, Mozes P. K. Blom

**Affiliations:** 1 Museum für Naturkunde, Leibniz Institute for Evolution and Biodiversity Science, Invalidenstraße 43, 10115 Berlin, Germany; 2 Faculty of Life Sciences, Humboldt-Universität zu Berlin, Unter den Linden 6, 10099 Berlin, Germany; 3 School of Natural Sciences, Macquarie University, Sydney, NSW Australia; 4 Department of Animal Biology, CE3C – Centre for Ecology, Evolution and Environmental Changes, Faculdade de Ciências da Universidade de Lisboa, University of Lisbon, Lisboa, Portugal; 5 Division of Ecology and Evolution, Research School of Biology, The Australian National University, Canberra, ACT Australia

**Keywords:** Evolution, Evolutionary biology

## Abstract

Organisms vary in their ability to cope with environmental perturbations, and even closely related species can differ in their resilience to climate change. For example, generalists may be better at accommodating environmental change than specialists with a narrow ecological niche. However, many species are difficult to classify as “specialist” or “generalist”, and may be merely adapted to distinct ecological niches. Furthermore, climate resilience may vary between ecological specialists if consequences are more profound for one ecological niche than another. In this study, we employ a multi-locus exon-capture approach and combine phylogeographic and population genetic methods to compare the evolutionary history of four species of Australian *Cryptoblepharus* lizards. These skinks co-occur in the Australian Monsoonal Tropics (AMT), have persisted despite major changes in Pleistocene climate, and have adapted to arboreal or rock substrates (two arboreal, two rock specialists). We find that phylogeographic structure is idiosyncratic between species and ecomorphs, likely shaped by the complex topography and heterogeneous environment of the AMT. In contrast, demographic analyses recovered largely congruent signals of expansion across populations, suggesting shared responses to past environmental change independent of ecomorph type. These results show that ecological specialization per se is not always a good predictor of demographic history or phylogeographic structure, and highlight the complex interplay between topography and climate history in promoting diversification. Thus, while ecological specialization, niche breadth, and other species-specific characteristics remain of interest, major landscape features that serve as biogeographic barriers or refugia may mask idiosyncratic responses between ecomorphs from the same adaptive radiation.

## Introduction

Climate is an important determinant of a species’ ecological niche, and changes in climate can have marked consequences for the distribution and demography of populations (Parmesan and Yohe 2003). There are three main responses of species to climate change: (i) species might be able to cope with changes in-situ, through phenotypic plasticity, changing their behavior or via local adaptation, (ii) they can mitigate changes by migrating to more suitable habitats, or (iii) species might be unable to cope with environmental change and may eventually go (locally) extinct (Davis et al. 2005; Hayden Bofill and Blom 2024). However, explicitly predicting species responses is not a trivial exercise, and studying past responses to major changes in the environment can provide valuable insights. Interestingly, a growing number of studies have highlighted that species responses can vary unexpectedly and may even differ substantially between closely related species (e.g., Bai et al. 2018; Fenker et al. 2021; Johnson et al. 2023; Leaché et al. 2020; Lim et al. 2017; Potter et al. 2018; Prates et al. 2016; Roycroft et al. 2024). One possible explanation could be that even closely related species can differ substantially in niche breadth, and species with a broad niche (generalists) may be more able to cope with changes in climate than species with a narrow niche (specialists) (e.g., Sexton et al. 2017; Carscadden et al. 2020). However, while recent studies have compared past population dynamics between generalists and specialists (Bell et al. 2010; Day et al. 2016; Afonso Silva et al. 2017; Oliver et al. 2019), this distinction may not be as conspicuous in the majority of organismal groups and is often context-dependent (Devictor et al. 2010). In many instances, species merely occupy a distinct ecological niche and may not necessarily differ much in terms of niche breadth or climatic tolerance (Devictor et al. 2010). It is therefore important to take ecological context into account and evaluate how co-occurring species adapted to a distinct ecological niche have responded to shared changes in past climate, particularly in biogeographic regions with a dynamic climatic history (Avise et al. 2016; Zamudio et al. 2016; Harvey et al. 2017; Edwards et al. 2022).

**Fig. 1:**
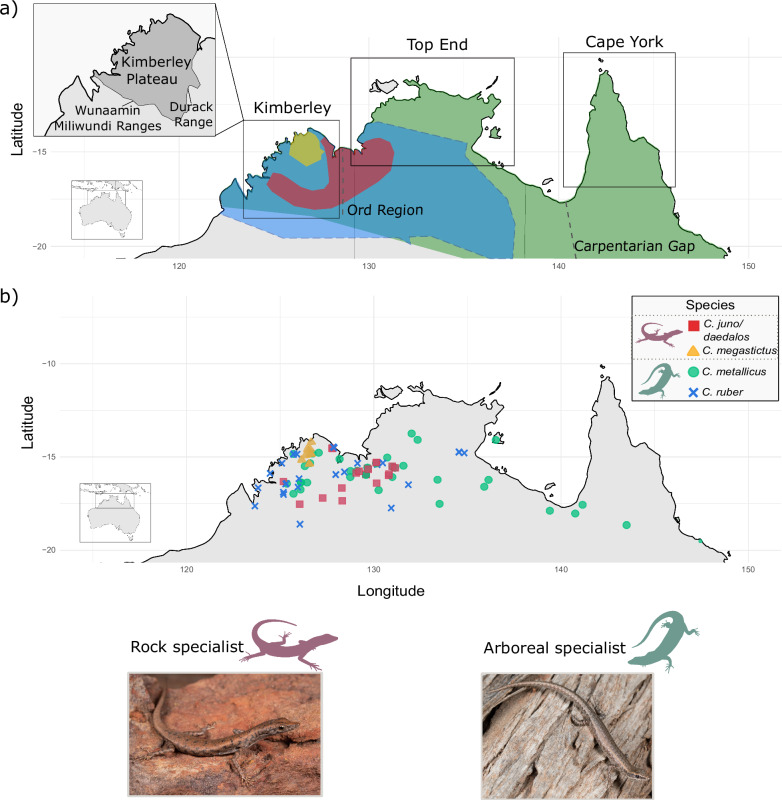
Regions in the Australian Monsoonal Tropics (AMT), distribution, and sampling of focal species. In (**a**), map of northern Australia displaying the three major regions contained in the AMT, major biogeographic barriers such as the Carpentarian Gap and the Ord Region, as well as the distribution of the four focal species taken from the Atlas of Living Australia (https://www.ala.org.au/) and the IUCN Red List of Threatened Species (https://www.iucnredlist.org/). In (**b**), sampling of the focal species used for exon-target capture. Photographs were taken by Stephen Zozaya and Scott Macor.

The Australian Monsoon Tropics (AMT, Fig. 1) is a large savanna woodland biome that is spread across northern Australia and is characterized by a heterogeneous landscape and complex climatic history (Bowman et al. 2010). The AMT can be divided into three biogeographic subregions: Cape York (Queensland, QLD), Top End (Northern Territory, NT) and Kimberley (Western Australia, WA) (refer to Bowman et al. 2010; Catullo et al. 2014; Edwards et al. 2017) (Fig. 1). During the Pleistocene, glacial cycles had a marked effect on aridity and monsoon duration, leading to the now well described expansion and contraction of savanna woodlands and, inversely, sandy deserts (Martin 2006; Reeves et al. 2013; Catullo et al. 2014). The dynamic interaction between climate and geography has played a major role in the diversification of many species and led to high levels of regional endemism observed in the AMT today (Potter et al. 2012, 2018, 2019; Catullo and Keogh 2014; Rosauer et al. 2016; Brennan and Oliver 2017; Edwards et al. 2017; Ashman et al. 2018; Oliver et al. 2022). Small skinks that belong to the genus *Cryptoblepharus* Wiegmann (Reptilia: Squamata: Scincidae), for example, rapidly diversified against this backdrop of climatic and environmental instability, and distinct species can be found throughout the AMT. *Cryptoblepharus* lizards are small diurnal skinks (~21–51 mm snout-vent length, Horner 2007) and they rapidly radiated across the Australian continent within the last five million years (Blom et al. 2016), mostly before the intensification of the climate cycles from one million years ago. Notably, during their radiation, *Cryptoblepharus* skinks repeatedly switched between substrates, and these independent shifts led to strong phenotypic convergence between distant species that inhabit the same habitat: Rock escarpments or tree trunks (Horner 2007; Cogger 2014; Wilson and Swan 2025). These distinct ecomorphs radically differ in several well-known adaptive traits, such as limb length and head height (Blom et al. 2016), but species that inhabit the same substrate are often highly cryptic, such that it is challenging to determine species identity based on morphological characters alone. As such, the diversification of Australian *Cryptoblepharus* represents a model system for understanding how both adaptive and non-adaptive processes can promote evolutionary radiations at a continental scale (Blom et al. 2016). Moreover, the persistence of distinct ecomorph pairs that co-occur in the AMT provides an exceptional opportunity to study concordance and incongruence in population dynamics between well-known ecological specialists that have faced major changes in shared climate.

In this study, we generate exon-capture data for a large number of *Cryptoblepharus* individuals that belong to two distinct species pairs, each consisting of a rock and arboreal ecomorph, that co-occur in the AMT, and investigate whether ecological specialization determines phylogeographic structure and past population dynamics in a region known for its dynamic climatic history. To address this question, we explicitly focused on species with overlapping distributions and targeted two ecomorphological species pairs that differ in their degree of divergence. The members of one species pair (*C. juno/daedalos* and *C. metallicus*) diverged a relatively long time ago (Blom et al. 2017), and they co-occur in all areas where *C. juno/daedalos* is found. While other species are perhaps more closely related to *C. juno/daedalos*, such species (e.g., *C. cygnatus*) have disjunct distributions and are endemic to geographic regions that likely differ slightly in climatic history. The other species pair (*C. megastictus* and *C. ruber*) are sister-species (Blom et al. 2016), diverged more recently from one another (~1–2 Myr) and co-occur in all areas where the rock specialist *C. megastictus* is found. All four species inhabit the Kimberley region in the western AMT but differ in the extent that they can be found in the greater AMT (Fig. 1). Notwithstanding these differences in geographic distribution or divergence time, we predict that species highly adapted to the same habitat are more similar in terms of phylogeographic structure and past population history, than closely related ecomorphs adapted to different habitats. For example, we expect that arboreal specialists have a more dynamic demographic history than rock specialists since Pleistocene aridification had a major impact on the distribution of savanna woodland (Byrne et al. 2008). At the same time, we expect that rock specialists harbor more intraspecific lineages than arboreal specialists due to their reliance on spatially isolated rocky ranges in which they may have been able to persist during past periods with an unfavorable climate. By studying ecomorph species in a comparative framework, we are not aiming to directly quantify the causal relationship between past climate and demographic history for each species alone, but ask whether the well-known Pleistocene cycles of aridification in the AMT (e.g., Potter et al. 2018; Roycroft et al. 2024) had a similar impact on species adapted to the same habitat and whether such signatures potentially differ between distinct ecomorphs.

## Material and methods

### Taxon sampling

We focused on two pairs of *Cryptoblepharus* species and compared the phylogeographic and demographic history between and within ecomorph pairs. The first pair includes the rock specialists belonging to the “Juno” complex (comprising *Cryptoblepharus juno* and *Cryptoblepharus daedalos*) and the widespread arboreal specialist *Cryptoblepharus metallicus*. In line with current taxonomy (Horner 2007), *C. juno* and *C. daedalos* are distinct species, but they are very closely related, have similar morphologies, and the narrow distribution of *C. daedalos* is largely enveloped by *C. juno* (Horner 2007). Since it remains questionable to what extent these are actually distinct species (Hayden Bofill et al. in Prep), for the purpose of the present study, we largely consider *C. daedalos* and *C. juno* as part of the same species complex (further quoted as “Juno”). Regardless, the distinct populations across all species are modeled independently. The second pair comprises the rock specialist *Cryptoblepharus megastictus* and the arboreal specialist *Cryptoblepharus ruber*. All four species can be found in the Kimberley, three have populations in the Top End, and only *C. metallicus* extends its distribution into Cape York (Fig. 1). Samples were sourced from our own field collecting (2012–2016), as well as from existing frozen museum collections to maximize geographic representation for all four species (Table S1).

We first sequenced a mitochondrial marker (NADH dehydrogenase 2—ND2, see SOM) for all individuals (*N* = 390). *Cryptoblepharus* species from the same habitat are notoriously difficult to discriminate based on morphology alone, and genetic examination using a mitochondrial marker provides an independent assay of species identity. Moreover, to date, none of these species have been screened with sequence data for phylogeographic diversity, and genetic differentiation has only been characterized using allozyme data with a select number of individuals (*C. juno* = 10; *C. daedalos* = 3; *C. megastictus* = 4, *C. metallicus* = 73, *C. ruber* = 22 individuals; Horner and Adams 2007). Particularly for the rock specialist *C. juno*, this early allozyme characterization only included the eastern part of their distribution. We then used an exon-capture approach to enrich target loci for each of the three species and the “Juno” species complex (25 “Juno” complex, 15 *C. megastictus*, 54 *C. metallicus*, and 31 *C. ruber*). The sampling density reflected the geographic distribution of each species and the lineage diversity recovered by the mitochondrial ND2 gene (Fig. S1). Finally, we also included three individuals from the species *C. leschenault* as an outgroup, which belong to an alternative clade of the focal species and only occur outside of Australia (the lesser Sunda islands, Indonesia; Horner 2007; Blom et al. 2019).

### Mitochondrial phylogeny

Following Sanger sequencing (see SOM), sequences were aligned and visually inspected in Geneious Prime v. 2021.2.2 (http://www.geneious.com/). We proceeded to infer a maximum-likelihood tree using RAxML (v.8.0; Stamatakis 2014). We selected the tree with the highest likelihood out of 100 replicates, assuming a general and time-reversible substitution model that allowed for rate heterogeneity among sites (GTR + Γ), and subsequently generated 1000 bootstrapped trees to estimate bipartition support across bootstrap replicates. As an independent validation of the phylogeny based on a single mitochondrial marker, we inferred a maximum-likelihood phylogeny with the 119 individuals for which we were able to recover largely complete mitogenome sequences from off-target exon-capture sequencing data (see SOM). For this, we employed ModelFinder Plus (Kalyaanamoorthy et al. 2017), implemented in IQTREE2 2.2.0.3 (Minh et al. 2020), to select the best substitution model for the data (GTR + F + I + G4) and reconstructed a maximum likelihood tree using the ultrafast bootstrap feature of IQTREE2 for 1000 bootstraps (Hoang et al. 2018).

### Nuclear phylogeny

#### Exon-capture

For the analyses based on nuclear loci, we used a targeted exon-capture approach for which exon sequences were generated using two different sequence capture designs based on exomes from related genera of skinks. An outline of the first capture approach can be found in Bragg et al. (2016), in which 3320 exons in 3226 different protein-coding genes were targeted, and the majority of genes were covered by a single exon (mean 1.03 per protein-coding gene). Our second design is a modified version of the original probe set, which targeted 2457 exons and excluded target regions that were not consistently recovered (Blom et al. 2019). Genomic libraries were prepared for 128 individuals using the protocol of Meyer and Kircher (2010) and modifications outlined in Bi et al. (2012). In short, we used ~1400 ng DNA per sample, and library preparation included blunt-end repair, adapter ligation, and adapter fill-in, followed by two separate index-PCRs to reduce PCR bias. All individuals enriched with the original capture design had a single unique barcode, whereas individuals targeted with the modified capture design had a double barcode combination. Barcoded libraries were pooled in equimolar ratios prior to hybridization, and the exon-capture hybridization was performed following the SeqCap EZ Developer Library user guide (Roche Nimblegen). We examined enrichment success using qPCR following the methods of Potter et al. (2019), which includes the use of specific primers to assess enrichment of targeted regions and de-enrichment of non-targeted regions of the genome. In addition, we measured the quantity and quality of library preparation pre- and post-capture using a Bioanalyzer (Agilent Technologies). Once the libraries passed all quality checks, they were submitted for sequencing (ACRF Biomolecular Resource Facility, Australian National University; Australia). We sequenced the enriched libraries (100 bp paired-end) on the Illumina HiSeq 2500 platform.

#### Sequence processing and alignment filtering

After sequencing, we cleaned the generated reads and removed low quality, low complexity and duplicate reads using a workflow developed by Singhal (2013). Briefly, exact duplicates due to PCR amplification were removed, and reads shorter than 36 bp long were discarded. Low complexity reads, which are reads with more than 60% missing data (“Ns”) or with homopolymer runs longer than 50% of read length, were excluded, and bases with average base quality below 20 across a 4-base window were trimmed. Cleaned reads were then mapped and assembled as described in Bragg et al. (2016; see SOM). To examine sequencing success and assembly quality for each individual, we calculated the number of exons recovered, the proportion of missing data (number of missing sites relative to total assembly length), and the proportion of heterozygous sites using Biopython (Cock et al. 2009) (Table S1). To minimize the possibility of spurious signals due to missing data, we used a conservative approach and only used individuals with more than 1000 exons sequenced. Furthermore, we removed individuals with a mean individual heterozygosity ratio above 0.01 (Figs. S2 and S3) to avoid inclusion of libraries that potentially contain cross-sample contamination and excluded samples with poor data quality and insufficient coverage. Based on these criteria, we retained a total of 94 individuals (19 “Juno” complex, 9 *C. megastictus*, 29 *C. ruber*, and 37 *C. metallicus*), with a mean depth of coverage, for each locus per individual, ranging between 22× and 358× (mean 112×). With consensus sequences in place, we then used a bioinformatics workflow explicitly designed for alignment and alignment filtering of exonic sequences, EAPhy V1.2 (Blom 2015). For analyses that only included a subset of individuals (e.g., individuals belonging to the same species), we reran EAPhy to optimize dataset-specific filtering, loci retention, and SNP selection. Dataset-specific filtering settings and more details on the EAPhy pipeline are all outlined in the SOM.

#### Phylogenetic inference

To reconstruct the evolutionary history between major lineages, we inferred a concatenated maximum-likelihood nuclear phylogeny with IQTREE2. Firstly, we assessed the consistency of the tree topology by inferring the nuclear tree with four different upper limits of missing individuals per locus (0, 5, 10, and 15) (Fig. S4). Results were largely concordant, and we hereafter only report findings based on the dataset that includes ambiguous sites and a maximum of 10 missing individuals per locus. We inferred a concatenated nuclear tree (1674 partitions and 834,115 total sites) and performed a partition model analysis (Chernomor et al. 2016), allowing IQTREE2 (ModelFinder Plus; Kalyaanamoorthy et al. 2017) to identify the substitution model with the best fit for each partition. Support values were calculated using the ultrafast bootstrap feature of IQTREE2 (Hoang et al. 2018) for 1000 bootstraps. Finally, we visualized the phylogenies in Figtree v.1.4.4 (http://tree.bio.ed.ac.uk/software/figtree/). All phylogenies were manually rooted using the *C. leschenault* individuals as outgroup.

### Population structure

We observed several instances of mitonuclear discordance across species (Fig. S5). Most surprisingly, the mitochondrial haplotype of *C. juno* and *C. daedalos* seems to be largely replaced by *C. metallicus* haplotypes. In other words, based on the mitochondrial phylogeny, the “Juno” complex is paraphyletic and nested almost entirely within *C. metallicus*. Therefore, we first applied several different approaches to the exon-capture data to explicitly test for possible cases of recent gene flow and admixture at both the interspecific and intraspecific level.

To characterize interspecific genetic clustering, we employed principal component analyses based on Euclidean distances (PCA) with the R package adegenet (Jombart 2008) for each pairwise combination of species (two comparisons: “Juno” complex (*N* = 19) × *C. metallicus* (*N* = 37) and *C. ruber* (*N* = 27) × *C. megastictus* (*N* = 9); Fig. S6). Second, we ran STRUCTURE v.2.3.4 (Pritchard et al. 2000) to uncover possible cases of admixture across the focal species pairs. For each species comparison, we conducted a STRUCTURE analysis using the admixture ancestry and independent allele frequency models for 20 iterations at *K* = 2 for the “Juno” complex × *C. metallicus* and, due to the paraphyletic placement of *C. ruber*, *K* = 3 for the *C. ruber* x *C. megastictus* comparison. We used a burn-in period of 100,000 generations and sampled an additional 200,000 generations afterwards. We used STRUCTURE Harvester v0.6.94 (Earl and vonHoldt 2012) to assess convergence of each run and to generate CLUMPP inputs, CLUMPP (Jakobsson and Rosenberg 2007) to align replicate runs, and Distruct v1.1 (Rosenberg 2004) to visualize the admixture proportions. For both PCA and STRUCTURE analyses, we repeated the analyses with alignments that included: (i) All available SNPs across loci or (ii) one randomly sampled SNP per locus. The former contains a much larger number of informative loci, but does not account for linkage between SNPs from the same exons.

Following the phylogenetic (mitochondrial and nuclear phylogenies) and interspecific clustering approaches (PCA and STRUCTURE), we proceeded to examine intraspecific population structure. For this, we employed the same STRUCTURE settings as for the interspecific comparisons, but this time we tested a range of different genetic cluster values per species (*K =* 1–5). Even though *C. megastictus* is nested within the lineage diversity of *C. ruber*; these two species were independently assessed for population structure, in accordance with the current taxonomy and the generally low support for the paraphyletic grouping of *C. ruber*.

In addition to STRUCTURE, we also used the R package Tess3r v1.1.0 (Caye et al. 2016), which utilizes geographically constrained matrix factorization and quadratic programming techniques (Figs. S7–S10). For *K* ranging between 1 and 5, we conducted each analysis using a genotype matrix and the geographic sample location. Since hierarchical clustering and isolation by distance (IBD) can lead to overestimation of the best *K*, we assessed the correlation between genetic distance and geographic distance using Mantel’s test with the R package dartR v2.7.2 (Gruber et al. 2018). In summary, we employed various approaches to test for possible signatures of past hybridization, characterize population structure within species, and to eventually assign individuals to their corresponding populations based on congruence between methods (e.g., phylogeny, PCA, STRUCTURE, IBD, and Tess3r).

### Demographic history

With population structure delineated for each species, we first calculated a range of population genetic parameters that are informative with respect to population history. We used the R package PopGenome v.2.7.5 (Pfeifer et al. 2014) to estimate absolute genetic diversity within (*π* and Watterson’s *θ*) and between populations (Dxy), using the same set of sites across species. We also calculated the net genetic diversity between populations (Da), as well as average heterozygosity per population (*H*_0_). We used the R packages rcompanion 2.5.0 (Mangiafico 2025) and FSA 0.9.6 (Ogle et al. 2025) to run a non-parametric one-way ANOVA (Kruskal and Wallis 1952) and post hoc Dunn tests with Benjamini–Hochberg (BH) correction (Dunn 1964) to compare estimates of mean heterozygosity across populations.

Demographic dynamics such as expansion and contraction have different effects on the resulting distribution of allele frequencies in populations. However, our analyses of population structure revealed substantial cryptic diversity and phylogeographic structure within most of the species. As a consequence, the number of individuals per genetic population was, in some cases, low, which hampered our ability to obtain a high-resolution estimate of allele frequencies. Moreover, while we used all individuals for the population structure analyses, we used a more restricted dataset for the demographic analyses to limit the possibility of a spurious demographic signal due to the inclusion of individuals with a recently admixed origin. The restricted dataset, therefore, excluded a total of 11 individuals that consistently showed signatures of putative admixture in the STRUCTURE and PCA plots, and with unexpectedly long branches in the maximum-likelihood phylogeny. For example, we excluded five *C. juno* individuals with 8–19% admixed ancestry in the STRUCTURE plot (Fig. S5) and three *C. metallicus* individuals that either had an admixed genomic composition, long branches, or were placed outside of the main *C. metallicus* clade. With a relatively limited sample size per population, we refrained from obtaining quantitative estimates of population history (such as population size or absolute divergence times) but rather focused on differentiating between three possible demographic scenarios: Expansion, contraction, and stable population size over time (Fraïsse et al. 2021). Moreover, we also evaluated whether allowing for gene-flow between populations resulted in a better model fit.

**Fig. 2:**
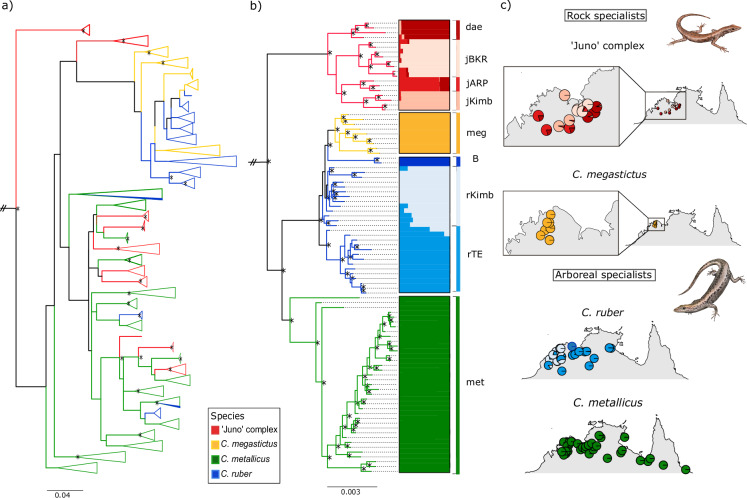
Phylogeographic and population structure of the four focal species. In (**a**), ND2 mitochondrial reconstruction including 390 individuals. In (**b**), a maximum-likelihood nuclear tree, bootstrap support over 95% is shown (*), and barplots depicting intraspecific analyses of population structure, including names of populations. In (**c**), admixture pie charts showing sampling across the AMT for arboreal and rock specialists. Illustrations of arboreal and rock ecomorphs are shown in (**c**). For clarity, the outgroup clade was removed.

Using the package PopGenome, we calculated Tajima’s *D* (Tajima 1989a, 1989b) for each population with a sample size *N* ≥ 5 using either all sites, only synonymous sites, or only non-synonymous sites (Table S5). We then ran 1000 coalescent simulations with the program MS (Hudson 2002) to simulate genetic samples according to a Wright-Fisher neutral model and evaluated whether the observed parameters differed significantly from neutrality (<0.05% of the simulated data). Tajima’s *D* values that deviate from zero can be interpreted as a signature of non-neutral processes acting on populations (Ramírez-Soriano et al. 2008; Ramos-Onsins and Rozas 2002; Tajima 1989a). In addition, we examined the evolutionary history of populations with an Approximate Bayesian Computation method for demographic inference, DILS (Fraïsse et al. 2021). DILS uses sequence data as input, accounts for linkage disequilibrium, and allows the joint inference of demographic history for up to two populations (Fraïsse et al. 2021). First, we ran single-population models for each population with five or more individuals to identify the best-fitting model out of three demographic scenarios (contraction, constant size, and expansion). Second, we used the two-population model to evaluate whether models that incorporate gene flow between populations fit the observed data better than strict isolation models. For this, we assessed four major demographic scenarios (strict isolation (SI), ancient migration (AM), isolation with migration (IM), and secondary contact (SC)). We only tested this for (i) species with more than one population, for (ii) pairwise combinations of populations between *C. ruber* and *C. megastictus*, since these species may have a paraphyletic history (Fig. 2), and (iii) between populations within the “Juno” species complex (i.e. *C. juno* and *C. daedalos*). For the one- and two-population DILS analyses, we only included loci with less than 50% missing data and a minimum length of 150 bp. To maximize the number of synonymous segregating sites for each population, we identified the minimum number of haplotypes retained per locus (“Nmin” filter) across a range of values (Tables S6 and S7). Priors included a generalized Squamate mutation rate of 6.125e-9 mutations per site and per generation (Gemmell et al. 2020), a generation time of 3.5 years, and a 0.1 ratio of recombination over mutation. For these analyses, no outgroup was included, and we therefore used the folded Site Frequency Spectrum (SFS). As priors, we ran an effective population size range of 50,000–20,000,000 individuals, a time of divergence between 5000 and 2,000,000 generations, and a migration rate range of 0.05–10, with a bimodal model for barriers. To improve model fit for some comparison pairs, we expanded the priors as follows: effective population size range of 50,000–40,000,000 individuals, time of divergence 100–2,000,000 generations, and migration rate range of 0.01–20. We allowed variable population size changes in each population. Finally, we ran five replicates per analysis to assess the convergence of results and used a custom-made R script to identify the best model and report the results for the replicate with the highest posterior probability (Potter et al. 2024). Goodness-of-fit as well as SFS plots for single- and two-population models are included in the Supplementary Material (Figs. S13–S16).

## Results

### Mitonuclear discordance

The phylogenetic patterns based on ND2 alone or whole mitochondrial genomes were largely congruent: (i) An unexpected lack of monophyly and paraphyletic clustering between individuals from different species, and (ii) clear phylogeographic structuring between populations (Figs. 2a and S1). To be more precise, mitochondrial clustering frequently matched geography rather than taxonomic classification. For example, in multiple instances, the mitochondrial haplotype of *C. juno* and *C. daedalos* (“Juno” complex) seemed to have been largely replaced with the haplotype of geographically nearby *C. metallicus* individuals (Figs. 2a, S1 and S5). In contrast, the nuclear maximum-likelihood phylogeny based on concatenation of 1674 filtered loci (Fig. 2b) provided strong support for the monophyletic clustering of individuals by species. The species *C. ruber* was the only exception, since *C. megastictus* was placed within the *C. ruber* clade, but its placement remained largely ambiguous (BS = 54). Nonetheless, there was strong support for the monophyletic grouping of samples from each of *C. megastictus* (BS = 100), the *C. ruber* NT lineage (BS = 100), the *C. ruber* “B” lineage (BS = 100), and moderate support for the *C. ruber* Kimberley lineage (BS = 95).

### Phylogeographic structure of rock and arboreal specialists

We used SNP data to detect any signal of admixture (interspecific species comparisons) and delineate population structure (intraspecific species analyses). Population genetic summary statistics were calculated using the same set of sites across populations (1,063,219 total sites; 11,182 total biallelic sites; 534–4723 segregating sites per population), whereas clustering and IBD analyses were conducted per species using either a single SNP per exon (1455–1813 SNPs) or all SNPs (6031–11,885 SNPs across all exons). Since both SNP datasets yielded similar results, here we only present the findings from the single SNP per locus datasets (but see Fig. S12 for clustering analyses using all SNPs). Clustering analyses based on allele frequencies were largely congruent with the nuclear phylogeny and recovered the four focal species and the corresponding intraspecific lineages.

#### Interspecific signatures of admixture

The STRUCTURE and PCA analyses conducted between species pairs were concordant with the nuclear maximum-likelihood phylogeny, and did not support a scenario involving widespread recent hybridization as an explanation for the extensive mitonuclear discordance (Fig. S5). In particular, five “Juno” individuals had little to moderate (8–19%) admixture with *C. metallicus*, and one *C. metallicus* individual from northern NT had 12% admixture with the “Juno” complex. These individuals were removed from downstream demographic analyses to avoid any possible spurious signatures due to recent admixture. In addition, a few other minor instances of possible admixture between species were recorded (Fig. S5). Two individuals of *C. ruber* from a remote location north-east of the Kimberley (“B”) had a 25% admixed origin and were excluded from one-population demographic models and Tajima’s *D* analyses. Similarly, three *C. megastictus* individuals from their northern Kimberley distribution also exhibited discernible signatures of admixture (9–21% admixture) with *C. ruber*; however, this signal was not detectable in the PCA, where in turn two different *C. megastictus* individuals were pulled apart from the rest of the species. These two individuals clustered within *C. ruber* in the mitochondrial phylogeny (Fig. S5) and were also excluded from downstream analyses. However, given the exhaustive survey undertaken, few individuals exhibited any signal of possible recent admixture, and the majority of individuals were unambiguously assigned to a unique population.

#### Intraspecific population structure

##### Rock specialists

**Fig. 3:**
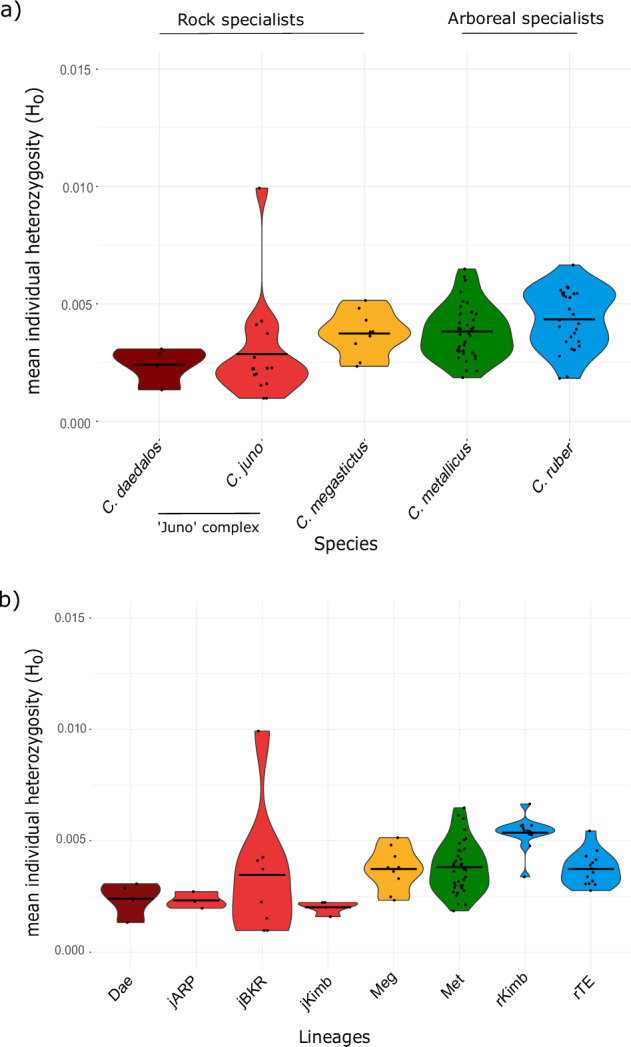
Genetic diversity of rock and arboreal specialists. In (**a**), mean individual heterozygosity per species, in (**b**), mean individual heterozygosity per lineage.

**Fig. 4:**
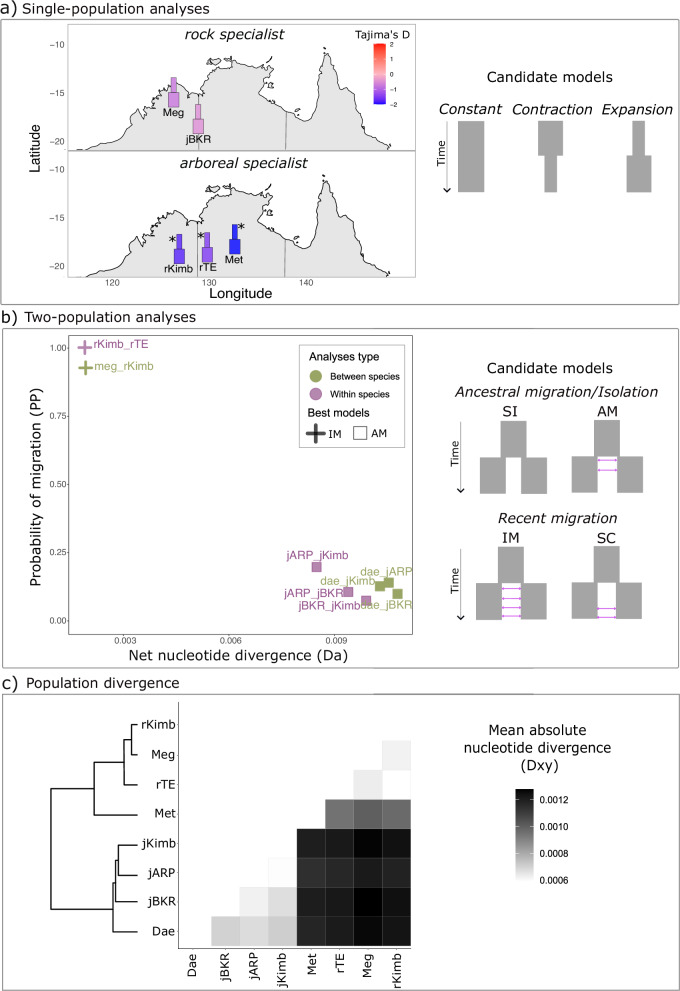
Demographic history of rock and arboreal specialists. In (**a**), the results of single-population analyses. Candidate models are illustrated on the right. The width and length of the models do not reflect any relative estimates. The best model is used as a symbol in the map, and Tajima’s *D* values are represented in the fill color. For a clearer representation, the *C. metallicus* population is shown in the middle of the AMT. Statistical significance of Tajima’s *D* is displayed as a star (*) next to the model symbol. In (**b**), the results of two-population models. Net nucleotide divergence (*D*_a_), probability of recent migration (as posterior probability) between lineages, and the best supported migration scenarios are shown. Lineage comparisons between species are illustrated in green and comparisons within species in purple. On the right, illustrations of the candidate migration models are shown. In (**c**), mean absolute nucleotide divergence (*D*_xy_) between lineages. The cladogram represents the nuclear phylogeny.

Visual inspection of STRUCTURE, Tess3r, and PCA results revealed largely congruent patterns and enabled the identification of four genetically distinct populations within the “Juno” complex: “Dae,” corresponding to the species *C. daedalos*, and three additional lineages, “jBKR,” “jARP,” and “jKimb” (Figs. 2 and S7). These four populations are distributed across geographically disjunct sandstone ranges or plateaus within the Kimberley and adjacent regions (Figs. 1 and 2c) and represent a substantial geographic extension of the known range for *C. juno* (Horner 2007; Cogger 2014; Wilson and Swan 2025). Moreover, notably, the degree of differentiation among the three populations of *C. juno* sensu stricto, as well as the genetic diversity within, is almost on par with the extent of divergence between *C. daedalos* and all other *C. juno* populations (Fig. 4c, Table S2). Due to strong phylogeographic structure within the “Juno” complex, the sampling density per population was more limited than anticipated. The IBD analyses were therefore only meaningful for the “jBKR” population and revealed a marginally positive correlation (IBD slope = 0.298, Mantel Test *p* = 0.023, Table S2). Within the “Juno” complex, the “jBKR” lineage displayed greater individual heterozygosity variation (H_0_-jBKR = 0.0035, Fig. 3b), but was not significantly different from the rest of the “Juno” lineages (Dunn-BH: *p* = 0.18–0.45; H_0_-jARP = 0.0023, H_0_-Dae = 0.0024, H_0_-jKimb = 0.002). The three other “Juno” lineages had, on average, a lower mean individual heterozygosity than *C. megastictus* and the arboreal lineages, but this was only statistically significant in comparison to the *C. ruber* “rKimb” population (Dunn-BH: *p* = 0.0001–0.0014; Fig. 3b, Table S2).

The species *C. megastictus* is endemic to the Kimberley region (Figs. 1 and 2c), and STRUCTURE, Tess3r, and PCA were somewhat incongruent when identifying the optimal number of populations (*K* = 1–3; Fig. S8). This incongruence may be partially explained by a strong signature of IBD (see *K* = 2 STRUCTURE and IBD; Fig. S8), suggesting that *C. megastictus* should be modeled as a single population (IBD slope = 0.301, Mantel Test *p* < 0.01, Table S2). In contrast to lineages within the “Juno” complex, genetic diversity estimates of the rock-adapted species *C. megastictus* were on par with mesic specialists; at both the species and population level (*H*_0_ = 0.0037, *θ*_W_ = 0.0006; Fig. 3, Table S2).

##### Arboreal specialists

The distribution of *C. metallicu*s spans the entire AMT (Figs. 1 and 2) and therefore also represents the largest sample size in our study (*N* = 34). Attempts to ascertain the optimal number of populations through various methods (Delta K, best likelihood, and cross-validation score) yielded incongruent results (Figs. S9 and S11). Notably, *K* > 1 reveals the underlying geographic substructure by displaying the genetic differentiation of the individuals between its eastern- and westernmost distribution (Fig. S9). The observed geographic substructure is driven by a strong signature of IBD (IBD slope = 0.671, Mantel Test *p* value = 0.001; Table S1, Fig. S9c). A distinct cluster representing three individuals from Queensland emerged in all analyses, including the PCA (Fig. S9a, b). Despite this differentiation, limited sampling from this region precluded its treatment as a separate population, and it is also geographically and ecologically disjunct from the rest of the species. Consequently, we characterized the Monsoonal Tropics population of *C. metallicus* as a single lineage that spans from the Kimberley region to the eastern Northern Territory (denoted as “Met,” Fig. 2). Notwithstanding its geographic spread, high connectivity and hence putatively large population size, the mean individual heterozygosity observed in *C. metallicus* (*H*_0_ = 0.0038; Fig. 3a, b, Table S2) is similar to the geographically restricted rock specialist *C. megastictus* and the populations of the arboreal specialist *C. ruber* (Table S2, Fig. 3a, b).

The arboreal specialist *C. ruber* is distributed across the NT and Kimberley, and coexists in sympatry or parapatry with all other focal species throughout various regions of its distribution (Fig. 1). The optimal number of genetic clusters ranged between 2 and 4 (Fig. S10). However, higher values of K showed no discernible spatial pattern (see *K* = 4 in Fig. S10a). Consequently, we identified *K* = 3 as the most suitable value (Fig. 2). Two primary genetic clusters were supported: (1) the Kimberley population (“rKimb”) and (2) a population that spans from eastern NT to the ranges surrounding the Kimberley (“rTE”) (Fig. 2b). The third genetic cluster comprises two individuals from a single site in the north-east of the Kimberley (“B,” individuals Crub387 and Crub366, Fig. 2a). Unfortunately, limited availability of samples from this location and admixture signatures with *C. megastictus* did not allow their inclusion in the demographic analyses. The two main populations, “rKIMB” and “rTE,” seem to have a contact zone located in central Kimberley (Fig. S10). Whereas no signal of IBD was observed for the “rKimb” lineage, IBD was observed within the NT lineage of *C. ruber* (IBD slope = 0.159, Mantel Test *p* value = 0.003; Table S2, Fig. S10c). We excluded one *C. ruber* individual from the demographic analyses with a nearly 50% admixture profile between the two *C. ruber* populations (individual “Crub793,” Fig. 2a). The mean individual heterozygosity level of *C. ruber* individuals is similar to that of the rock specialist *C. megastictus* and arboreal specialist *C. metallicus* (Table S2, Fig. 3a, b).

### Demographic history of rock and arboreal specialists

To compare the population history of the four species and corresponding populations, we first assessed signatures of recent expansion, contraction, or constant size by estimating Tajima’s *D* and single-population models for populations with at least five individuals. Tajima’s *D* and one population model results were largely concordant, but not always statistically significant; namely, the Tajima’s *D* values for the rock specialists (Tables S4, S5, S8 and S10). Beyond the single-population models, we also assessed the genetic connectivity between populations for species with more than one distinct genetic cluster, where phylogenetic relationships between species remained ambiguous (i.e., *C. ruber* and *C. megastictus*) or with unclear taxonomy (i.e., “Juno” complex) (Tables S9 and S11). Models with recent or ancestral gene flow were preferred over strict-isolation models, across all intra- and interspecific comparisons with two-population models (posterior probability = 0.80–1.00; Table S9).

#### Rock specialists

For the *C. juno* “jBKR” (*N* = 10 haplotypes) population, Tajima’s *D* estimates were negative across all datasets (all sites included, synonymous sites or non-synonymous sites only; Table S5), but none were statistically significant. The DILS single-population models consistently supported an “expansion” model (Table S6), with a posterior probability >0.99 for almost all replicates. Unfortunately, the sampling for the other *C. juno* populations did not suffice (*N* < 5) for inferring demographic history on a per-population basis. Ancestral migration models were supported over recent migration or strict isolation models for all pairwise combinations of “Juno” populations (posterior probability = 0.68–0.76; Fig. 4, Table S9). Moreover, no difference in migration probability was observed when comparing *C. daedalos* and the other *C. juno* lineages, providing further support for our notion to treat *C. daedalos* as part of the *C. juno* species complex within the context of the present study.

The rock specialist *C. megastictus* formed a single population, and population history was therefore modeled based on all *C. megastictus* individuals that passed filtering requirements (*N* = 7). Similar to *C. juno* (“jBKR”), Tajima’s *D* was negative but not statistically significant for all datasets (all sites included, synonymous sites or non-synonymous sites only; Table S5) while all replicates for the DILS single-population model strongly supported an expansion model (posterior probability = 1.0, Tables S4 and S8). Interestingly, the probability of recent migration was higher between these distinct ecomorphs (posterior probability = 0.94) than between populations that belong to the same putative species (see *C. juno* populations, for example). This result aligns with the low genetic divergence observed between *C. megastictus* and the sympatric *C. ruber* population (“rKimb”) (*D*_xy_ = 0.0006, *F*_st_ = 0.11; Fig. 4; Table S3).

#### Arboreal specialists

The demographic analyses of *C. metallicus* (*N* = 50 haplotypes) presented a consistent signature of population expansion (Table S4), with a strongly negative and statistically significant Tajima’s *D* (−2.232), and support for population expansion across all replicates of the DILS one-population model (posterior probability = 0.98, Table S8). Notwithstanding its widespread distribution, *C. metallicus* is best characterized as a single population across the WA-NT portion of the Monsoonal tropics, and we therefore did not estimate population connectivity or calculate migration probability between distant sampling localities.

Similar to *C. metallicus*, the demographic analyses supported a history involving population expansion for the two major *C. ruber* lineages (*N*_rKimb_ = 22 haplotypes; *N*_rTE_ = 22 haplotypes), as indicated by a negative Tajima’s *D* (*C. ruber* – “rKimb”: −1.797, *C. ruber* – “rTE”: −1.527) and the DILS one-population models (“rKimb”: posterior probability = 1.0, “rTE”: posterior probability = 0.99). The DILS two-population model for *C. ruber* converged on a history of recent migration and a relatively high migration probability between the Top End and Kimberley populations (posterior probability = 1.0).

## Discussion

Glacial cycles during the Pleistocene had a major effect on the flora and fauna of the AMT. Against this backdrop of climatic instability (Reeves et al. 2013), *Cryptoblepharus* skinks persisted, and their adaptive radiation sets the stage for a comparative study between closely related but ecologically divergent species that co-occur across large areas of the AMT and have a shared climatic history. For this purpose, we first assayed phylogeographic diversity across 100s of individuals using a single mitochondrial marker and then used sequence capture to obtain 1000s of orthologous nuclear loci for 125 individuals. Our study is the first comprehensive characterization of phylogeographic diversity using sequence data within any *Cryptoblepharus* species and yielded some surprising new insights. The extent of population structure within species differed as much within as between ecomorph categories, while population diversity statistics and demographic models recovered somewhat congruent signals of expansion across all populations, regardless of ecomorph type.

### Phylogeographic structure and biogeography

Notwithstanding strong phenotypic convergence between species adapted to the same substrate (Blom et al. 2016), the extent of phylogeographic structure within both rock and arboreal ecomorphs differs substantially (Fig. 2a, b). Within the “Juno” complex, we identified four major lineages while *C. megastictus* only comprises a single population. The four “Juno” lineages, including the currently recognized *C. daedalos*, are consistently supported across analyses and display clear genetic differentiation across their distribution (Fig. 2). Moreover, we identified two individuals from a north-eastern locality that may belong to an additional (5th) population, but further sampling is needed. In contrast, *C. megastictus* only comprises a single population that spans most of the north-central Kimberley region (Fig. 2). The contrasting phylogeographic structure between *C. megastictus* and the “Juno” complex may scale to some extent with divergence time, since the “Juno” complex appears to have diverged slightly earlier from its sister species than the more recently formed *C. megastictus* (Blom et al. 2017). However, the distribution (Fig. 2), mean individual heterozygosity (Fig. 3), and IBD analyses (Fig. S8) all suggest that *C. megastictus* represents a large panmictic population that is spread across a relatively wide geographic range, whereas populations within the “Juno” complex tend to be more restricted to isolated ranges at the periphery of the Kimberley. Thus, even when taking divergence time into account, the evolutionary dynamics shaping phylogeographic structure within *C. megastictus* seem to differ quite substantially from lineages within the “Juno” complex.

The extent of phylogeographic structure also differs between the two arboreal species. The species *C. metallicus* has the largest distribution amongst nearly all *Cryptoblepharus*, but clustering analyses only recovered a single population across most of the AMT, and the underlying population structure is congruent with an isolation-by-distance model. The arboreal specialist *C. ruber*, on the other hand, has a smaller geographic distribution but does harbor two discernible lineages with a parapatric distribution: (i) A Kimberley population and (ii) a Northern Territory population. Yet, despite their genetic differentiation, these two populations come into contact towards the center of the Kimberley, and we recorded hybrids with mixed ancestry in these contact zones (Fig. S10). Furthermore, for both *C. ruber* and *C. metallicus*, we identified a few individuals that may belong to distinct populations (a putative Queensland population for *C. metallicus* and a divergent north-eastern population for *C. ruber*), but sampling was too limited to make informed conclusions about their status or to include them in the demographic analyses. Notwithstanding the same ecological requirements, overlapping distributions across the range of *C. ruber*, and no difference in divergence time (Blom et al. 2017), *C. ruber* differed from *C. metallicus* with a more pronounced underlying population structure at a smaller spatial scale.

Rather than matching by ecomorph category, the distribution of lineage diversity across the landscape seems to align more closely with known biogeographic barriers and echoes phylogeographic patterns observed in other taxa. The species *C. megastictus* is a Kimberley restricted rock specialist, and *C. ruber* also includes a population that is primarily confined to the Kimberley (“rKimb”) region. Similarly, the topological and topographical heterogeneity of the Kimberley region has likely also played an important role in determining population structure within the “Juno” complex, with populations having narrow distributions around the periphery of the central Kimberley. These phylogeographic patterns are consistent with other taxa (Afonso Silva et al. 2017; Fenker et al. 2021; Moritz et al. 2018; Oliver et al. 2010, 2012; Potter et al. 2018), including rock specialists (Pepper et al. 2011; Potter et al. 2012; Laver et al. 2018; Oliver et al. 2019; Zozaya et al. 2023). Major geological divisions in the Kimberley include the Kimberley Plateau in the center and the highly complex Wunaamin Miliwundi and Durack Ranges on the southern and eastern edge (Pepper and Keogh 2014). The contrasting topographic features of these divisions have likely led to notable distinctions in the phylogeographic structure of many taxa across the Kimberley, where it is not uncommon to find genetically distinct lineages occupying the periphery and the north-central Kimberley plateau (e.g., Laver et al. 2018; Moritz et al. 2018; Zozaya et al. 2023). The rock specialists in this study match this pattern, with *C. megastictus* being solely found in the north-central Kimberley plateau, whereas the distinct populations of the “Juno” complex are distributed across the distinct ranges on the periphery. The escarpments surrounding the Kimberley could have either served as a hard barrier, preventing lineages from invading each other’s range, or the current distribution is a status quo, and competition between identical ecomorphs prevents further changes in biogeographic distributions. However, interestingly, while the phylogeographic distribution of *C. ruber* also follows this trend, this pattern in phylogeographic structure is completely absent within *C. metallicus*, which stretches across most of the AMT from eastern NT to the Kimberley coast. It remains unclear what prompts the phylogeographic delineation at the Kimberley border within *C. ruber* but not in *C. metallicus;* two species that are morphologically cryptic and seemingly identical in habitat requirements.

In addition to the distribution of lineage diversity for each species, we also uncovered a surprising extent of mitonuclear discordance between species and across ecomorphs. Most notably, the *C. juno* mitochondrial haplotype is almost entirely replaced by a *C. metallicus* copy, but these patterns are not mirrored in the nuclear genome. Species are largely monophyletic in the nuclear phylogeny, and few individuals show moderate levels of recent admixture between species. These findings suggest that hybridization may have solely taken place early in the diversification of the genus (“ancestral hybridization”) or that selection against nuclear introgression is very strong. Mitonuclear discordance has also been observed for other taxa across the AMT, especially in the Kimberley, and correlates with the dynamic climatic history of the region, leading to expansion and contraction, and secondary contact between populations (Moritz et al. 2016; Laver et al. 2018; Doughty et al. 2018; Moritz et al. 2018; Potter et al. 2024). Broader sampling and genomic coverage is needed to distinguish between adaptive and neutral processes as a mechanism driving this mitonuclear discordance. However, interestingly, mitonuclear discordance is mostly apparent between species that are distinct ecomorphs (e.g., *C. juno* vs. *C. metallicus*, and *C. megastictus* vs. *C. ruber*) and is rarely recovered between highly cryptic species that use the same habitat. These findings raise important questions regarding the prevalence and evolutionary implications of interspecific hybridization and the mechanisms facilitating species recognition (e.g., Zozaya et al. 2019).

### Demographic history

The inference of demographic history can yield important insights into the evolutionary processes that have shaped the contemporary phylogeographic and population diversity across a given landscape (Edwards et al. 2022). However, a representative sampling of current populations is needed to accurately model past population dynamics (McLaughlin and Winker 2020). While the accuracy of parameter estimation increases with sample size, model identification is still feasible with low sample sizes (*N* = 4; Robinson et al. 2014). To our surprise, our exon-capture dataset uncovered a substantial degree of cryptic diversity, particularly for the “Juno” complex. Consequently, our sampling density per population was relatively uneven, with many representatives for *C. metallicus* (*N* = 34) and few samples (*N* < 5) for three *C. juno* populations, preventing us from accurately estimating Tajima’s *D* or running DILS models across all populations. In addition to sample size, it is also important to note that the use of coding regions may, in some cases, lead to biased demographic estimates (Johri et al. 2021). However, other genome-reduction strategies also come with caveats (e.g., non-random missing data, non-orthologous loci, etc.) that may equally bias demographic inference. At the onset of the present study, no high-quality reference genome was available for *Cryptoblepharus*, and the costs associated with whole-genome resequencing were relatively high. We therefore opted for a genome-reduction strategy that yielded: (i) A large number of orthologous loci across all species, (ii) high-coverage for each locus, and (iii) cost-efficient to be employed at a phylogeographic scale for multiple species. Equivalent exon datasets have been shown to provide estimates of demographic history consistent with those from more widespread genomic sampling (SNP and whole genome datasets in koalas; Lott et al. 2022, 2024; McLennan et al. 2025). Moreover, excluding or including synonymous or non-synonymous substitutions had virtually no effect on our estimates of Tajima’s *D* (Table S5). Notwithstanding the unanticipated extent of cryptic diversity and the use of exonic loci, several trends emerged that contrast with the incongruence in phylogeographic patterns observed between species belonging to the same ecomorph category.

The species *C. metallicus* and *C. ruber* have both adapted to an arboreal habitat, have mostly overlapping distributions, and both consistently showed strong signals of population expansion. For the two *C. ruber* populations, Tajima’s *D* and the one-population DILS model supported scenarios of population expansion (Fig. 4). The observed signature of gene flow between the two *C. ruber* lineages, in both the clustering analyses and model-based inference, highlights a history of gene flow despite the presence of major biogeographical barriers surrounding the Kimberley (Pepper and Keogh 2014) and distinct evolutionary trajectories. At the eastern end of their distribution, the arboreal specialists differ in the extent of their geographic range (Rosauer et al. 2018). The Carpentarian Gap, located between the QLD and NT regions, has been acknowledged as a biogeographic barrier leading to population isolation and genetic divergence in other savanna vertebrates (Ford and Blair 2005; Lee and Edwards 2008; Bowman et al. 2010; Catullo et al. 2014; Eldridge et al. 2014; Kearns et al. 2014; Pepper et al. 2017; Edwards et al. 2023) and could also have prevented the eastward expansion of *C. ruber* into Queensland. The lack of such a barrier for *C. metallicus* could potentially be explained by a different timing of expansion. Notably, during the Pleistocene, Cape York and the Top End were intermittently connected due to marine regressions (up until 40,000 years ago) and continuously connected until ~10,000 years ago (Chivas et al. 2001; Ford and Blair 2005), providing an opportunity for some species to cross into Queensland across the Carpentarian Plain. The Carpentarian Gap may therefore differ in strength over time and lead to contrasting patterns of genetic structure (Eldridge et al. 2014). Nonetheless, it remains to be tested whether population structure arising from periods of isolation between marine regression could have been erased by the periodic gene flow following the re-emergence of the Carpentarian Plain. Alternatively, perhaps *C. metallicus* and *C. ruber* do have fine-scale differences in habitat requirements that are not easily discernible with our present understanding of the ecology of these species.

Among rock specialists, we could only compare *C. megastictus* to one of the three *C. juno* populations (“jBKR”), due to the prevalence of cryptic diversity and the corresponding limited sampling per population. *C. megastictus* represents a relatively large Kimberley population that is in isolation-by-distance equilibrium, and we observed either strong statistical support (DILS; Table S8) or a tendency with a negative Tajima’s *D* (Table S5), for an expansion scenario. Similarly, the “jBKR” population represents the distribution of *C. juno* across the Bullo River and Keep River National Parks, and also shows strong statistical support (DILS; Table S8) or a tendency (Tajima’s *D*; Table S5) for an expansion scenario. Despite a low sample size per “Juno” complex population, we ran two-population DILS models for a preliminary view on population connectivity between distinct mountain ranges. These inferences should be interpreted with caution due to their low sample size (*N* < 5), but in combination with the presence of strong phylogeographic structure, the various *C. juno* populations and *C. daedalos* have likely persisted in their isolated respective mountain ranges over long timescales (Fig. 4). However, more exhaustive sampling is certainly needed for all populations within the “Juno” complex and may resolve the taxonomic status of the various populations. More specifically, regardless of sequence divergence, these preliminary models did not recover any meaningful difference in terms of migration probability between *C. daedalos* and within the various *C. juno* populations (Fig. 4b).

Pleistocene aridification had a major effect on the distribution of savanna woodland, and we hypothesized that this dynamic history would be echoed in the demographic past of arboreal specialists. At the same time, we expected that the demographic history of rock specialists would be less dynamic due to their reliance on habitats that are geographically fixed, though topographically heterogeneous, increasing the likelihood of persistence in such regions. Our findings only partially supported these hypotheses. We indeed found strong support for population expansion, regardless of method (i.e., Tajima’s *D* or DILS), across all arboreal populations. Moreover, *C. metallicus*, one of the most widely distributed species of *Cryptoblepharus*, has very limited phylogeographic structure across a large area of the AMT; further supporting the notion that it may have expanded relatively recently and rapidly. However, contrary to our expectations, demographic inference for the two rock specialists also seems to support expansion scenarios, albeit without statistical support for Tajima’s *D* analysis. The marginal incongruence between the DILS and Tajima’s *D* analysis can be interpreted as support for an expansion model, but the strength of population expansion is likely much lower than for the arboreal specialists. Additional sampling of individuals and loci could put this hypothesis further to the test, by moving beyond merely comparing demographic models to estimating model parameters themselves as well (e.g., Ne, divergence time, etc.). Either way, our present findings suggest that all populations seem to have expanded to some extent and that ecomorph type may only be somewhat indicative of the magnitude of expansion.

## Conclusion

While most studies have compared generalist versus specialist species, few studies have explicitly asked whether specialist species adapted to similar environments are congruent in their response to past changes in climate. Here, our findings indicate that deep phylogeographic structure within species did not correlate with habitat specialization but seems to correspond more closely with known biogeographic barriers, though with some exceptions, such as *C. metallicus*. At the same time, habitat specialization did not serve as a good predictor of recent demographic patterns and was not necessarily correlated with ecomorph type. However, due to the high degree of cryptic diversity, sampling per population was limited, and future research should put this hypothesis to the test with more dense sampling per population, a larger number of species pairs, and ideally using whole-genome resequencing data. With such a dataset in place, future studies can examine how the demographic tendencies described for the ecomorphs ultimately translate into the idiosyncratic phylogeographic structure observed for species adapted to the same habitat. This will improve our ability to better understand the role of climate, landscape, and species-specific characteristics in facilitating climate change resilience of individual species and how this relates to long-term evolutionary change.

## Supplementary information

Supplemental Information

Table S1

## Data Availability

ND2 and target-capture sequences are publicly available in NCBI (National Center for Biotechnology Information) under the accession numbers PQ155520 - PQ155900 and under the Bioproject PRJNA1171859, respectively (Table S1 in Appendix). Mitochondrial sequences, scripts, and other relevant data are available in the Dryad repository 10.5061/dryad.x3ffbg801.
