# Supplementary material for: Diversification across the Australian Monsoonal Tropics: Comparing phylogeographic and demographic patterns within and between species of Cryptoblepharus skinks
Source: Heredity (Edinb). 2026 Apr 18;135(6):409–21. doi: 10.1038/s41437-026-00843-8 (PMC13354775; doi:10.1038/s41437-026-00843-8)
Supplement: Supplementary file 1 — Supplemental Information [file 41437_2026_843_MOESM1_ESM.docx]

**Supplemental Information for:**

**“Diversification across the Australian Monsoonal Tropics: Comparing phylogeographic and demographic patterns within and between species of *Cryptoblepharus* skinks”**

**Table of Contents:**

| **Supplementary Methods** | Page 2 |
| --- | --- |
| **Figure S1** | Page 6 |
| **Figure S2** | Page 7 |
| **Figure S3** | Page 8 |
| **Figure S4** | Page 9 |
| **Figure S5** | Page 10 |
| **Figure S6** | Page 11 |
| **Figure S7** | Page 12 |
| **Figure S8** | Page 13 |
| **Figure S9** | Page 14 |
| **Figure S10** | Page 15 |
| **Figure S11** | Page 16 |
| **Figure S12** | Page 17 |
| **Figure S13** | Page 18 |
| **Figure S14** | Page 19 |
| **Figure S15** | Page 20 |
| **Figure S16** | Page 21 |
| **Table S1** | Attached as excel file/in Dryad |
| **Table S2** | Page 23 |
| **Table S3** | Page 24 |
| **Table S4** | Page 25 |
| **Table S5** | Page 26 |
| **Table S6** | Page 27 |
| **Table S7** | Page 29 |
| **Table S8** | Page 31 |
| **Table S9** | Page 32 |
| **Table S10** | Page 33 |
| **Table S11** | Page 34 |

**Supplementary Methods**

1. **DNA extraction and mitochondrial sequencing**

We extracted DNA using the salting-out method of Sunnucks & Hales, (1996). We amplified the ND2 segment using the PCR primer (5’ – 3’): L4437b - AAGCAGTTGGGCCCATRCC, and ND2R_102 – CAGCCTAGGTGGGCGATTG (Smith et al. 2007) or ND2_f_jA – CACTCATACTAACTAACCTTGC, and ND2_r_jB - GTCTATCTAGGAGGCTTTAGC. PCRs were performed in 25 μL reactions containing ~100 ng DNA, 2.5 μL 10x PCR buffer, 0.2 mM dNTP, 2.5 mM MgCl2, 10 pmol each forward and reverse primer and 0.5 U Taq DNA Polymerase (Invitrogen). Each PCR reaction was run on a Corbett PC-960C cooled thermal cycler: initial denaturation step of 94 °C (1 min.), followed by 38 cycles of 94 °C (45 sec.), 52 °C (45 sec.) and 72 °C (1 min.). Each PCR run finished with a final extension period of 1 min. at 72 °C. We purified PCR products with an exonuclease sequence clean-up (5 μl of PCR product, 0.4 μl Exonuclease 1, 1.6 μl Shrimp Alkaline Phosphatase and 3 μl of distilled water) at 37 °C for 45 min. followed by 80 °C for 15 min. Purified PCR products were sequenced in 20 μl reactions containing 0.8 μl BigDye Terminator v3.1 (Applied Biosystems), 4.5 μl 5x sequencing buffer, 3.2 pmol primer, 1 μl purified PCR product and 13.5 μl double-distilled water (see Potter et al. 2016 for Big Dye cycle sequencing conditions). We washed the products with sodium acetate and eluted them in 20 μl of HiDi formamide, prior to sequencing on an ABI 3100 DNA analyzer.

In addition to ND2 sequence data, we also reconstructed whole or partial mitochondrial genomes assembled from ‘by-catch’ sequencing reads (non-target sequenced reads) from the 128 individuals included in the exon capture experiment. Briefly, a *Cryptoblepharus* specific mitochondrial reference genome was recovered using an iterative baiting and mapping approach; MITObim (Hahn et al., 2013). We generated a *Cryptoblepharus* specific draft reference genome based on reads from a species outside of the focal clade (*C. zoticus*; Blom et al., 2016b) by using an annotated mitochondrial genome of the skink *Lygosoma sundevalli* as an initial bait. We then mapped polished sequencing reads (see below) for each exon-capture library to the reference genome, called variants and a consensus sequence. We used BWA-mem v0.7.17 (Li, 2013) to map reads, sorted reads by coordinates and converted SAM to BAM files with Picard v2.20.7 (https://broadinstitute.github.io/picard/) and called variants with Freebayes v1.3.1 (Garrison & Marth, 2012). For variant calling, only reads with a mapping quality above 20 were used and variants with a quality score below 20 or allelic balance between 0 and 0.2 were ignored. We used VCFfilter v0.1 (https://biopet.github.io/vcffilter/0.2/index.html) to remove indels and flagged heterozygous positions. Biallelic mitogenome sites could be a signal for contamination or NUMTs and were routinely masked as a precaution. Sites below 5x or above 1000x coverage were masked as well. Consensus sequences for each individual were called with GATK FastaAlternateReferenceMaker v4.1.1.0 (Van der Auwera & O’Connor, 2020). Assembled mtDNA genomes were then checked for the overall proportion of missing sites. Mitogenomes with less than 35% missing data were aligned using MAFFT v7 (Katoh et al., 2002; Katoh & Standley, 2013; Table S1) and alignments were visually inspected using GENEIOUS Prime v. 2021.2.2 (<http://www.geneious.com/>).

1. **Exon-capture**

For the first capture design, exon-targets were obtained based on orthologs from seven transcriptomes of three genera closely related to *Cryptoblepharus* (*Carlia rubrigularis*, *Lampropholis coggeri*, and *Saproscincus basiliscus*; Singhal, 2013). The modified design included transcriptomes from the aforementioned species as well as exonic information of a subsequently sequenced *Cryptoblepharus ruber* (Blom et al., 2016). Briefly, target exons were identified with a balanced base composition (30—70% GC content) in the genome of *Anolis carolinensis* (Ensembl release 67, Flicek et al., 2014) and selected orthologs that were also present in the transcriptomes of the skink species mentioned above. A total of 3,320 exons were targeted in the first capture design (each exon greater than 200 base pairs in length) and 2,457 in the second design (Bragg et al., 2016; Blom et al., 2019). The targeted exons were synthesized into a sequence capture probe set (SeqCap EZ Developer Library) by Roche NimbleGen.

1. **Sequence processing and alignment filtering**

Cleaned reads were mapped and assembled as described in Bragg et al., (2016). Briefly, cleaned reads were initially mapped to the original exons used for target design and libraries were subsequently individually assembled using Velvet (v.3; (Zerbino & Birney, 2008), K = 31, 41, 51, 61, 71, 81). The assembled contigs from different K values were merged with CAP3 (Huang & Madan, 1999) and trimmed to their respective exon boundaries using EXONERATE (v.2.2; Slater & Birney, 2005). We discarded contigs that covered less than 65% of the length of the target exon. If multiple contigs were assembled, we applied a reciprocal best blastx hit criterion to select orthologous to the targeted *Anolis* protein (Blom et al., 2016; Bragg et al., 2016). We then used the assembled contigs for each individual as its own reference and mapped cleaned reads back to these best contigs. Mapping was performed using BOWTIE2 (v.2.2.2; Langmead & Salzberg, 2012) and resulting SAM files processed with SAMTOOLS (v.0.1.19; Li et al., 2009). We employed GATK (McKenna et al., 2010) to identify heterozygous sites (with a minimum sequencing depth of 16x, Bragg et al., 2016), mask sites with a low-quality genotype call (GQ<20) and generated phased haplotypes using the individual sequencing reads (‘read backed phasing’).

Filtering and alignment of exon sequences for each individual were done with EAPhy V1.2 (Blom, 2015). In brief, sequences were aligned with MUSCLE v3.8.31 (Edgar, 2004), individual sequences with gaps with more than three amino acids in a seven-codon window within an alignment were trimmed and individual sequences with more than one stop codon were removed. To filter out codon columns with missing data, we removed alignments in which more than three amino acids in a seven-codon window differed from the alignment consensus. Finally, only alignments over 150 bp in length were considered for further analysis. EAPhy generates a set of different outputs: i) alignments for individual filtered exons, ii) a concatenated alignment of all filtered exons, iii) a concatenated alignment of all polymorphic sites, and iv) two replicates of a concatenated alignment where a single SNP was randomly sampled for each exon, including only biallelic and parsimony informative sites. The concatenated alignment including all filtered exons was used for phylogenetic analyses and ABC demographic inference with DILS, while the concatenated SNP alignments were used for population genetic analyses.

**References**

Auwera, G. A. V. der, & O’Connor, B. D. (2020). *Genomics in the Cloud: Using Docker, GATK, and WDL in Terra*. O’Reilly Media, Inc.

Blom, M. P. K., Bragg, J. G., Potter, S., & Moritz, C. (2016). Accounting for uncertainty in gene tree estimation: Summary-coalescent species tree inference in a challenging radiation of Australian lizards. *Systematic Biology*, syw089. <https://doi.org/10.1093/sysbio/syw089>

Bragg, J. G., Potter, S., Bi, K., & Moritz, C. (2016). Exon capture phylogenomics: Efficacy across scales of divergence. *Molecular Ecology Resources*, *16*(5), 1059–1068. <https://doi.org/10.1111/1755-0998.12449>

Edgar, R. C. (2004). MUSCLE: Multiple sequence alignment with high accuracy and high throughput. *Nucleic Acids Research*, *32*(5), 1792–1797. <https://doi.org/10.1093/nar/gkh340>

Evanno, G., Regnaut, S., & Goudet, J. (2005). Detecting the number of clusters of individuals using the software structure: A simulation study. *Molecular Ecology*, *14*(8), 2611–2620. <https://doi.org/10.1111/j.1365-294X.2005.02553.x>

Flicek, P., Amode, M. R., Barrell, D., Beal, K., Billis, K., Brent, S., et al. (2014). Ensembl 2014. *Nucleic Acids Research*, *42*(D1), D749–D755. <https://doi.org/10.1093/nar/gkt1196>

Garrison, E., & Marth, G. (2012). *Haplotype-based variant detection from short-read sequencing* (arXiv:1207.3907). arXiv. <https://doi.org/10.48550/arXiv.1207.3907>

Hahn, C., Bachmann, L., & Chevreux, B. (2013). Reconstructing mitochondrial genomes directly from genomic next-generation sequencing reads—A baiting and iterative mapping approach. *Nucleic Acids Research*, *41*(13). <https://doi.org/10.1093/nar/gkt371>

Huang, X., & Madan, A. (1999). CAP3: A DNA Sequence Assembly Program. *Genome Research*, *9*(9), 868–877. https://doi.org/10.1101/gr.9.9.868

Katoh, K., Misawa, K., Kuma, K., & Miyata, T. (2002). MAFFT: A novel method for rapid multiple sequence alignment based on fast Fourier transform. *Nucleic Acids Research*, *30*(14), 3059–3066. <https://doi.org/10.1093/nar/gkf436>

Katoh, K., & Standley, D. M. (2013). MAFFT Multiple Sequence Alignment Software Version 7: Improvements in Performance and Usability. *Molecular Biology and Evolution*, *30*(4), 772–780. <https://doi.org/10.1093/molbev/mst010>

Langmead, B., & Salzberg, S. L. (2012). Fast gapped-read alignment with Bowtie 2. *Nature Methods*, *9*(4), 357–359. <https://doi.org/10.1038/nmeth.1923>

Li, H., Handsaker, B., Wysoker, A., Fennell, T., Ruan, J., Homer, N., et al. (2009). The Sequence Alignment/Map format and SAMtools. *Bioinformatics*, *25*(16), 2078–2079. <https://doi.org/10.1093/bioinformatics/btp352>

Li, H. (2013). Aligning sequence reads, clone sequences and assembly contigs with BWA-MEM (arXiv:1303.3997). *arXiv*. <https://doi.org/10.48550/arXiv.1303.3997>


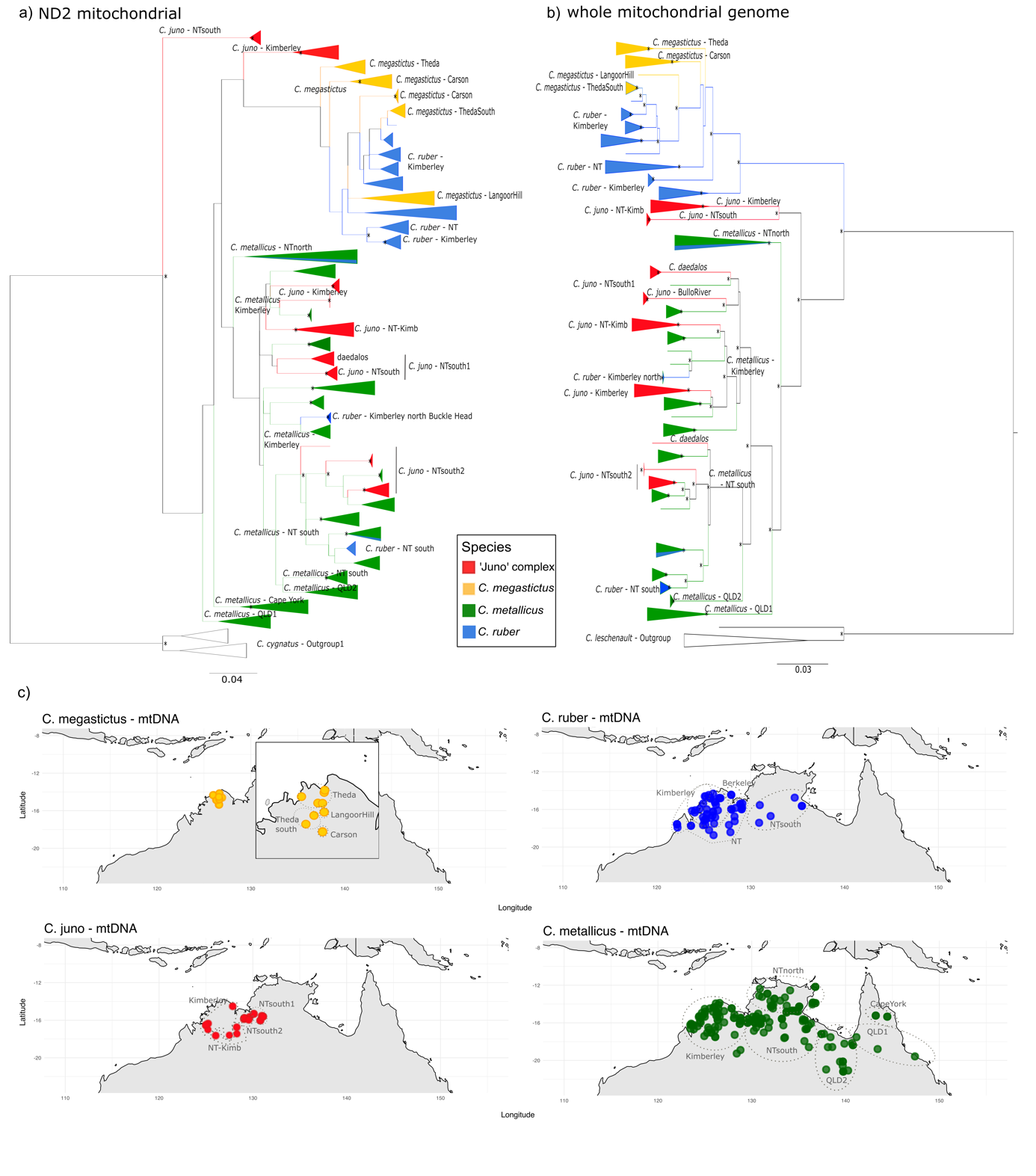


Figure S1. Maximum-likelihood phylogenies for the mitochondrial ND2 and whole mitochondrial genome. Major phylogeographic lineages are identified within a) the mitochondrial ND2 and b) within the whole mitochondrial genome phylogeny. Species are characterized by colors with ‘Juno’ complex in red, *C. megastictus* in yellow, *C. ruber* in blue and *C. metallicus* in green. Bootstrap support over 0.85 are marked with an *.


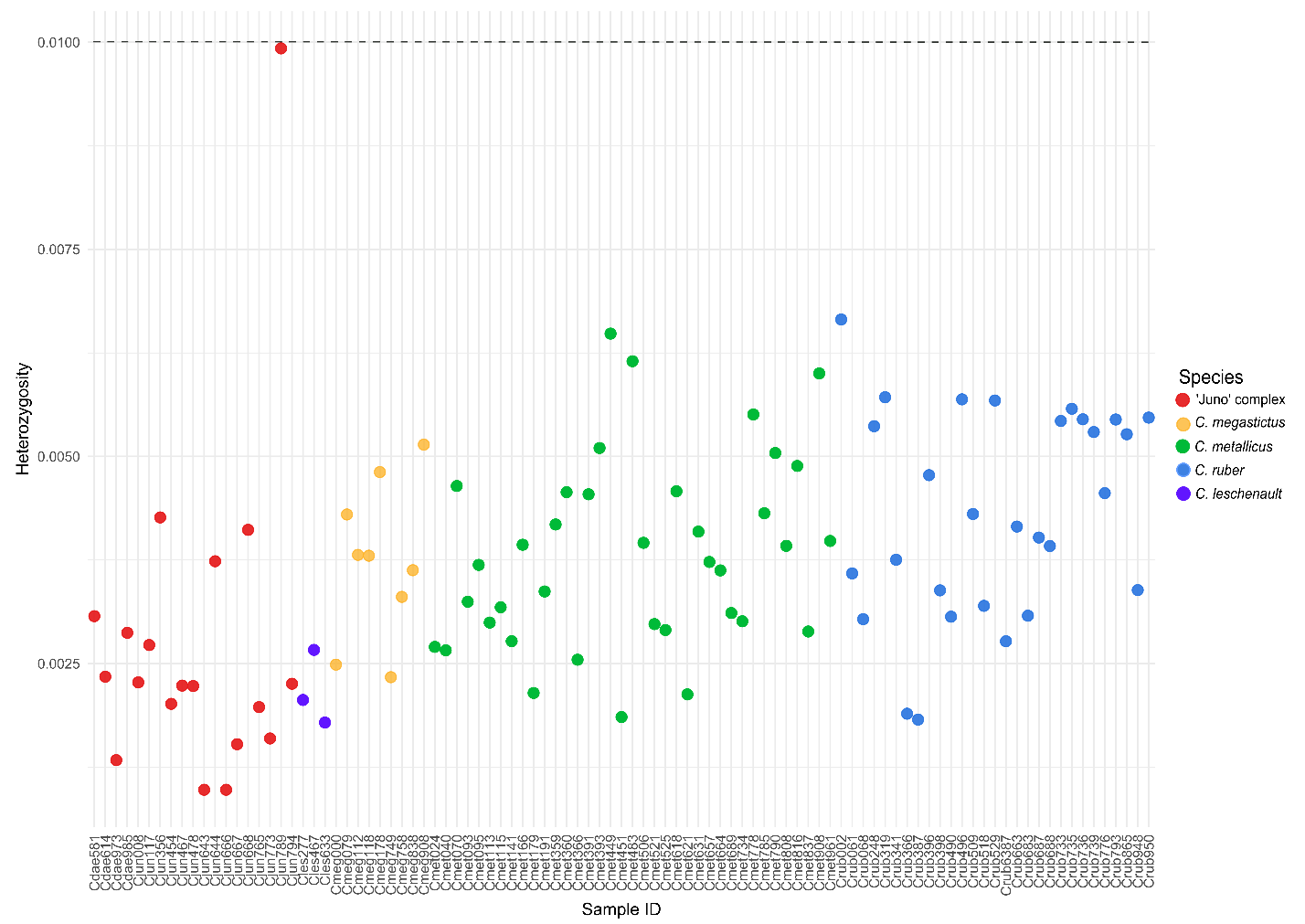


Figure S2. Mean individual heterozygosity. Mean heterozygosity levels of the 97 sequenced individuals (including three outgroup individuals) that passed the filtering criteria and that were included in downstream analyses. Individuals are colored by species and cut-off of 0.01 is shown in a dashed line. Three *C. leschenault* individuals used as outgroup are included.


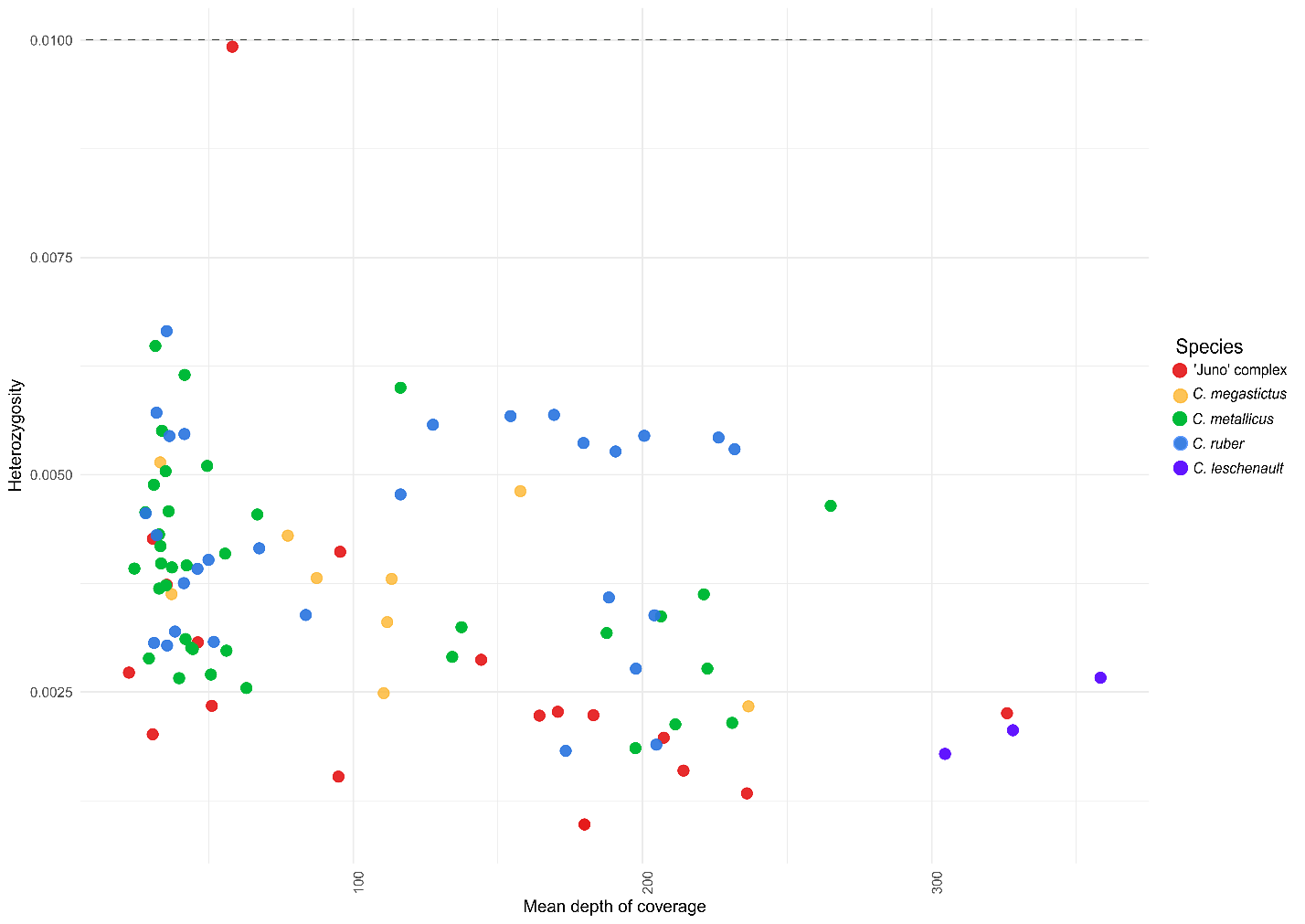


Figure S3. Mean individual heterozygosity against mean individual depth of coverage. Values are shown for the 97 sequenced individuals (including outgroup individuals) that passed filtering criteria and were included in downstream analyses. Individuals are colored by species and cut-off of 0.01 is shown in a dashed line.


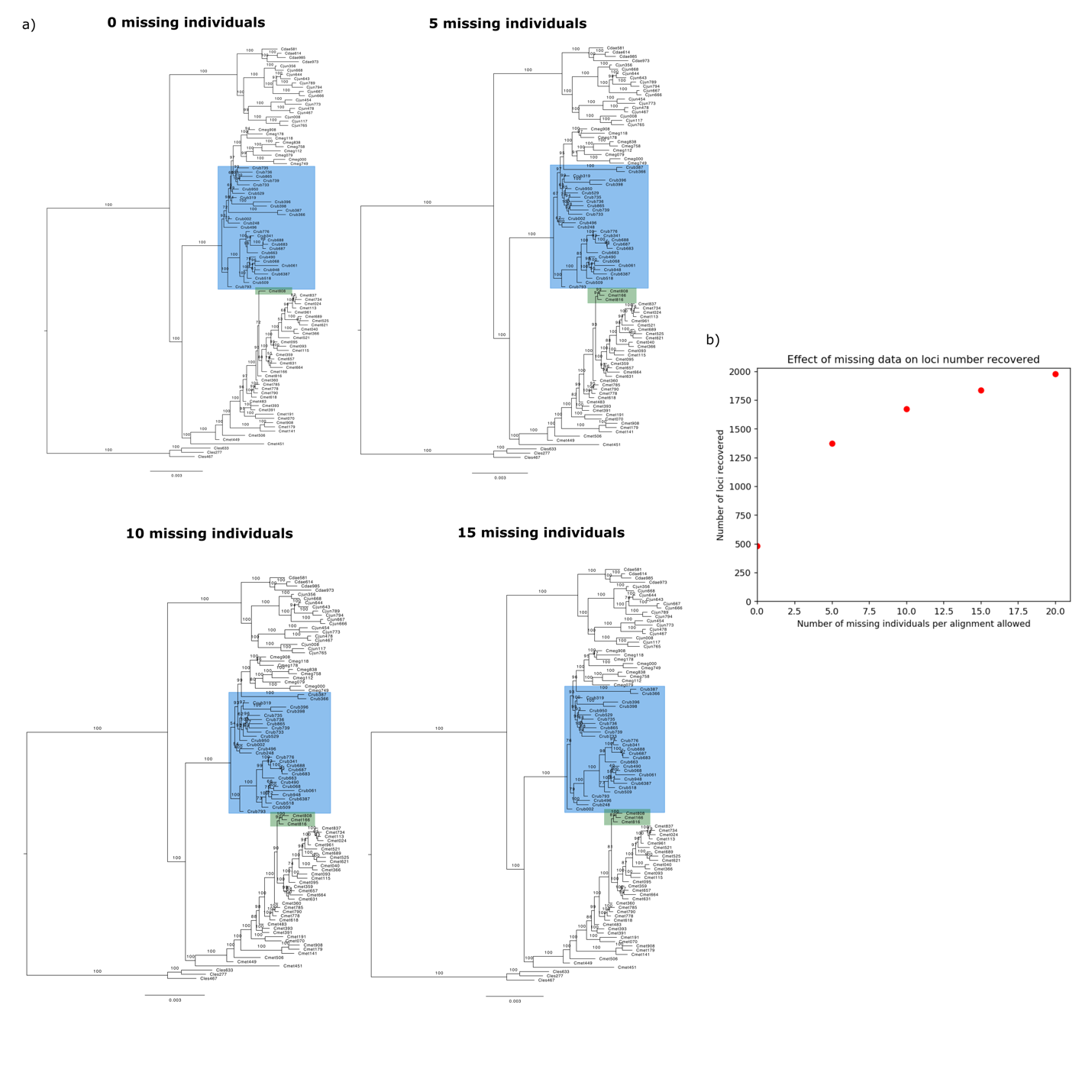


Figure S4. Consistency of nuclear phylogeny across various levels of missing individuals per locus. In a) four nuclear topologies generated with different levels of missing individuals (0, 5, 10 and 15 missing individuals) are shown. Highlighted areas in blue and green show variations in the phylogenetic positioning of some *C. ruber* and *C. metallicus* individuals, respectively. In b), number of loci recovered across different levels of missing individuals is displayed.


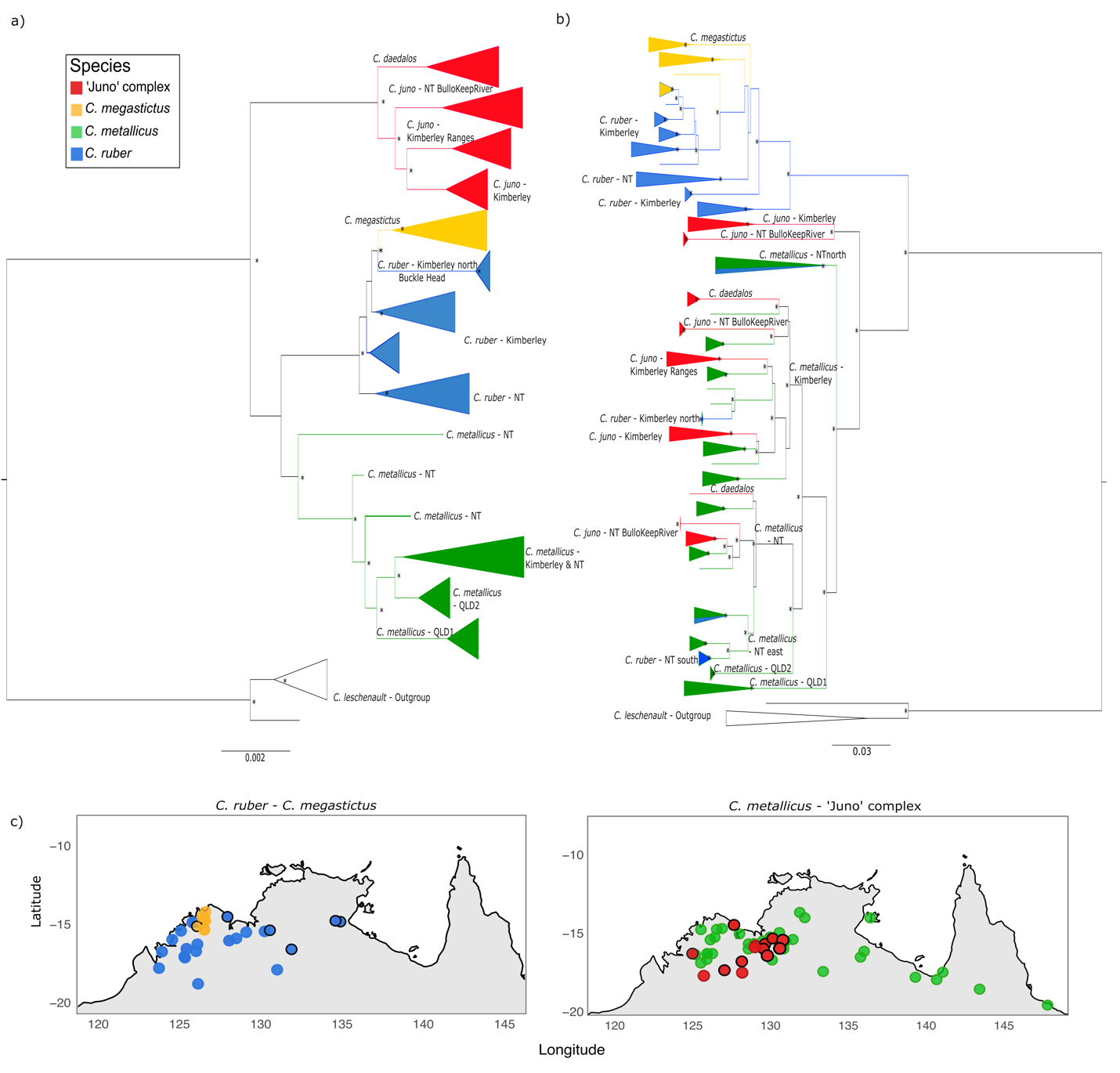


Figure S5. Mitonuclear discordances based on targeted exon-capture between three species and one species complex of *Cryptoblepharus* lizards in the AMT. In a) nuclear (left) and whole mitochondrial (right) genome phylogenies are shown. The four focal species are displayed in different colors (see legend). Three *C. leschenault* are used as an outgroup. Bootstrap support over 0.85 are displayed with an *. In b), mitonuclear discordances are shown based on the geographic location of individuals for each of the species comparisons (*C. ruber* – *C. megastictus* on the left, *C. metallicus* – ‘Juno’ complex on the right). Fill colors represent the nuclear identity whereas stroke color shows the mitochondrial identity of the individual.


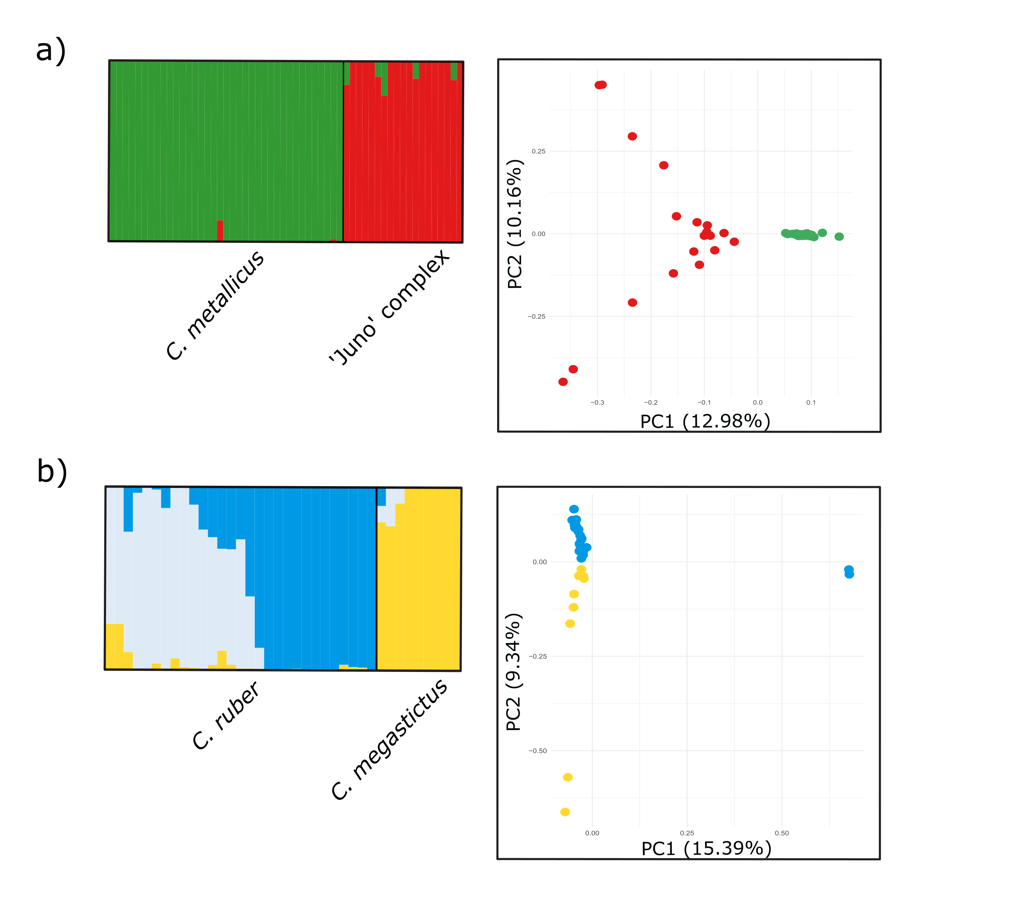


Figure S6. Species pair comparisons. PCA and STRUCTURE analyses are shown for the two interspecific comparisons. In a), K=2 was used to assess admixture levels between the ‘Juno’ complex and *C. metallicus*, whereas in b) K=3 was specified to assess admixture between *C. megastictus* and *C. ruber*.


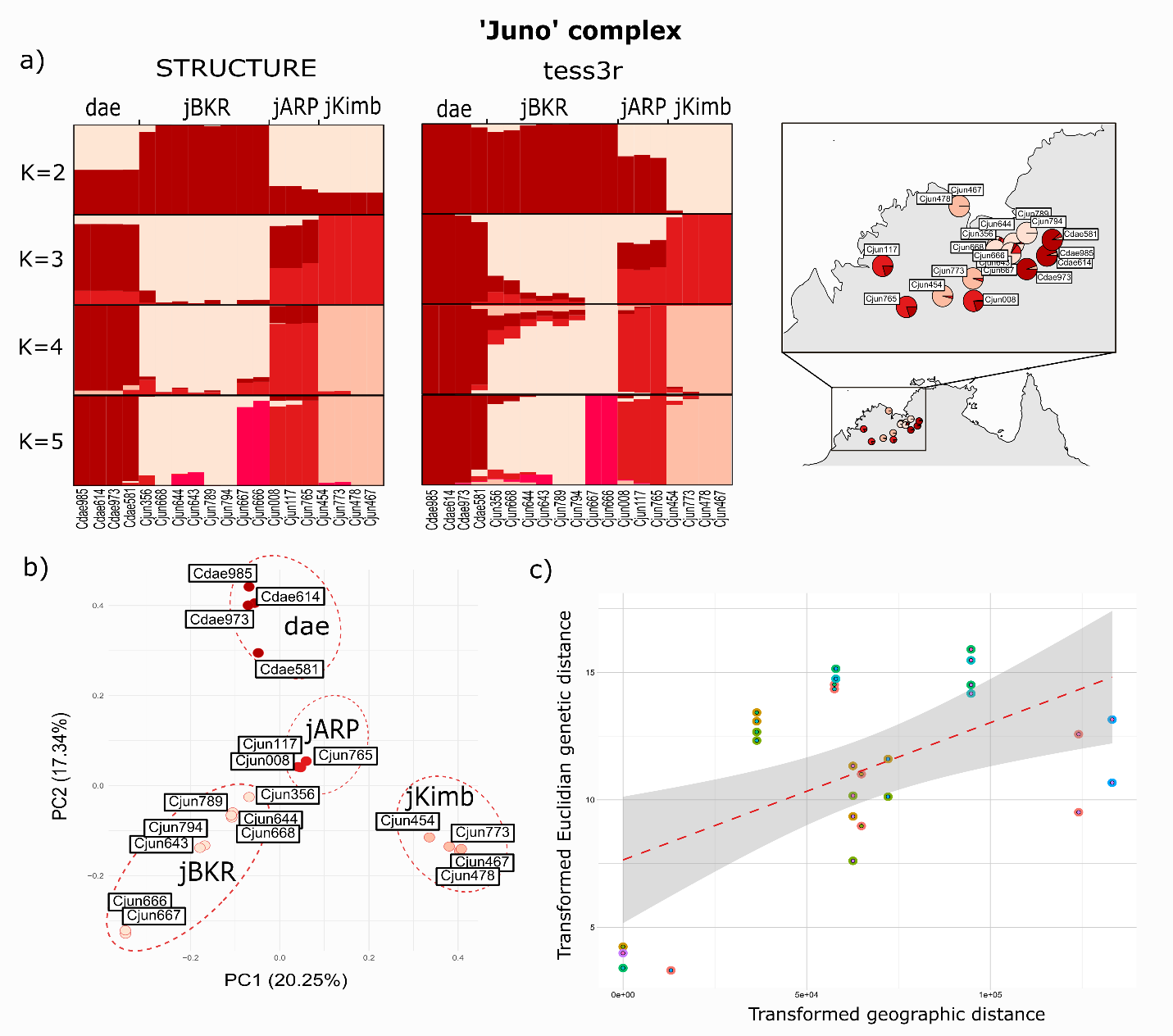


Figure S7. Intraspecific population structure analysis of the rock specialist ‘Juno’ complex. In a) results of STRUCTURE and Tess3r analyses for K values ranging from two to five. The map shows admixture proportions for the best K selected for *C. juno* (K = 4). PCA results are shown in b), and c) displays the slope from the isolation by distance test for the ‘jBKR’ population.


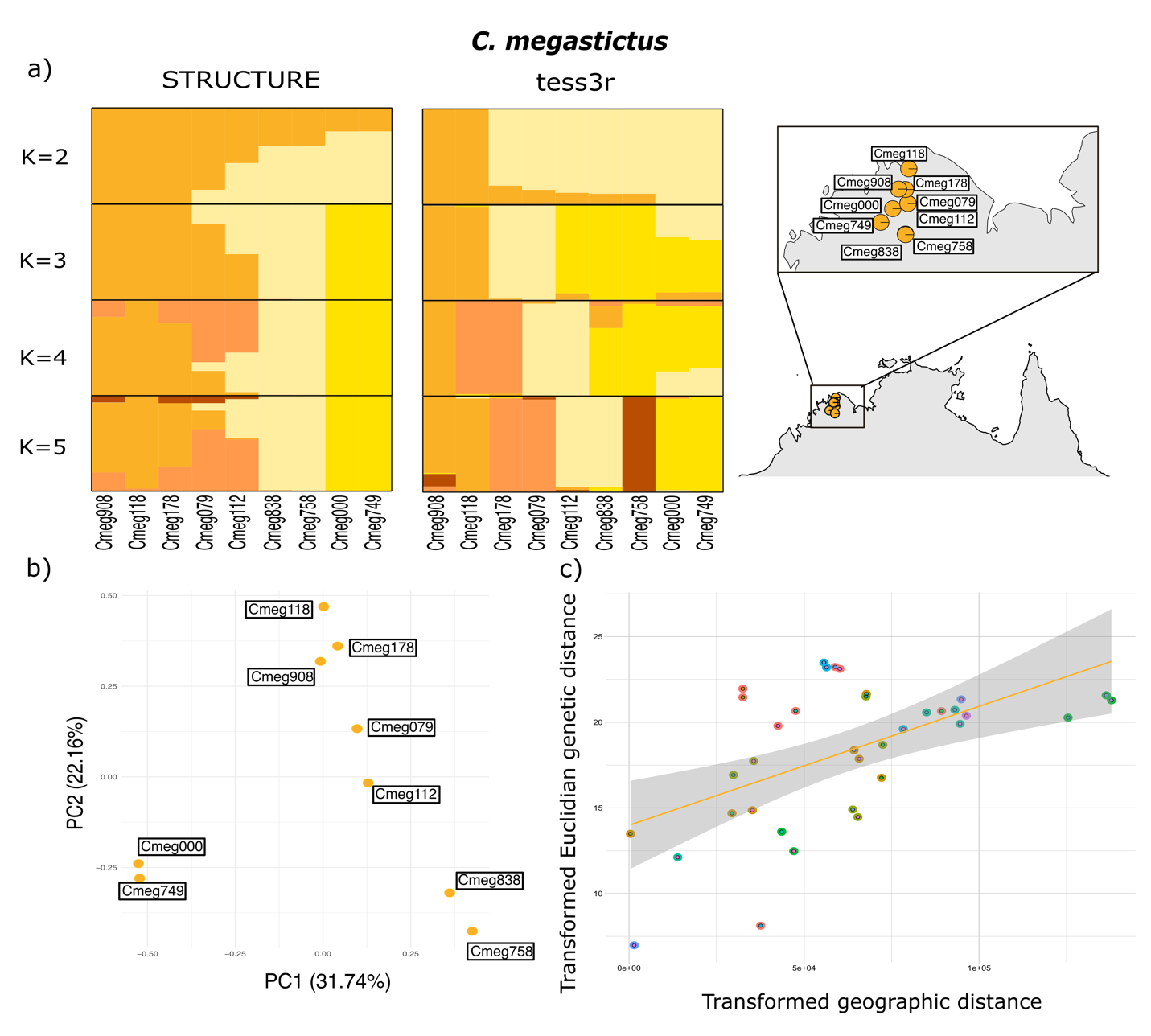


Figure S8. Intraspecific population structure analysis of the rock specialist *Cryptoblepharus megastictus*. In a) results of STRUCTURE and Tess3r analyses for K values ranging from two to five. The map shows admixture proportions for the best K selected for *C. megastictus* (K = 1). PCA results are shown in b), and c) displays the slope from the isolation by distance test for the species.


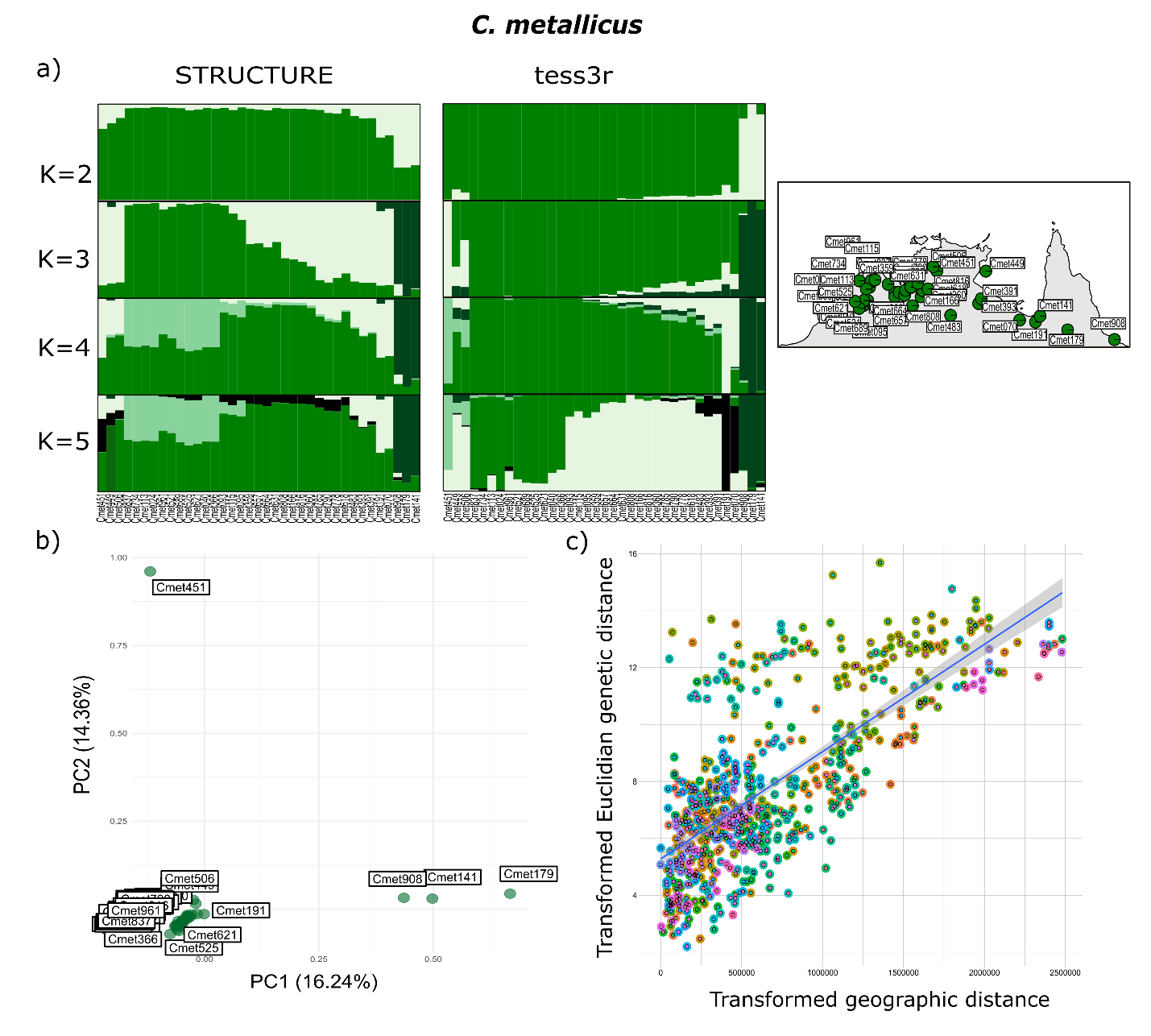


Figure S9. Intraspecific population structure analysis of the arboreal specialist *Cryptoblepharus metallicus*. In a) results of STRUCTURE and Tess3r analyses for K values ranging from two to five. The map shows admixture proportions for the best K selected for *C. metallicus* (K = 1). PCA results are shown in b), and c) displays the slope from the isolation by distance test for the species.


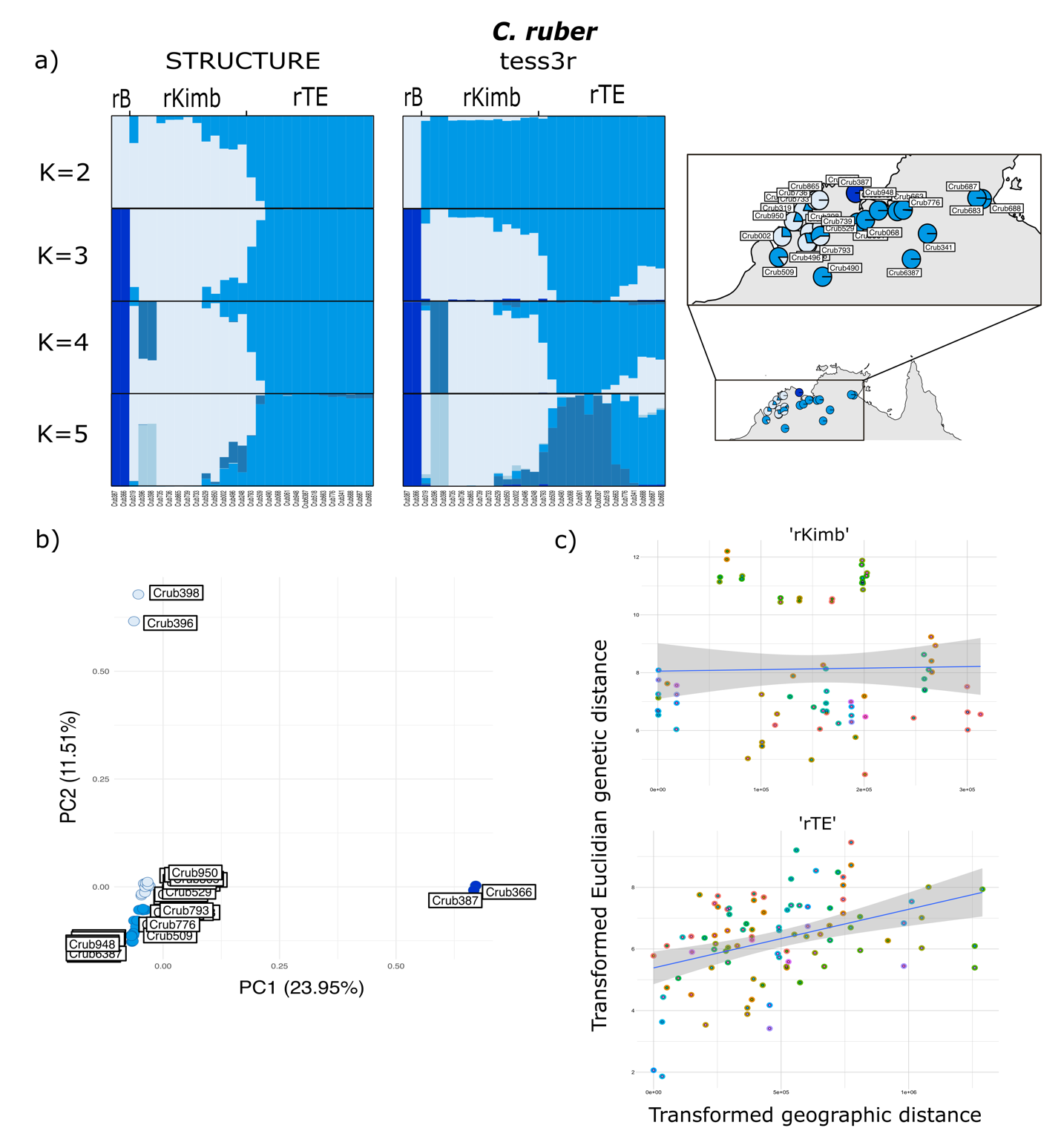


Figure S10. Intraspecific population structure analysis of the arboreal specialist *Cryptoblepharus ruber*. In a) results of STRUCTURE and Tess3r analyses for K values ranging from two to five. The map shows admixture proportions for the best K selected for *C. ruber* (K = 3). PCA results are shown in b), and c) displays the slope from the isolation by distance test for the species.

Figure S11. Best genetic cluster (K) value supported by the Evanno method (Evanno et al., 2005) and best likelihood approaches for STRUCTURE, and cross-validation scores for Tess3r. These analyses were done using one SNP per exon.


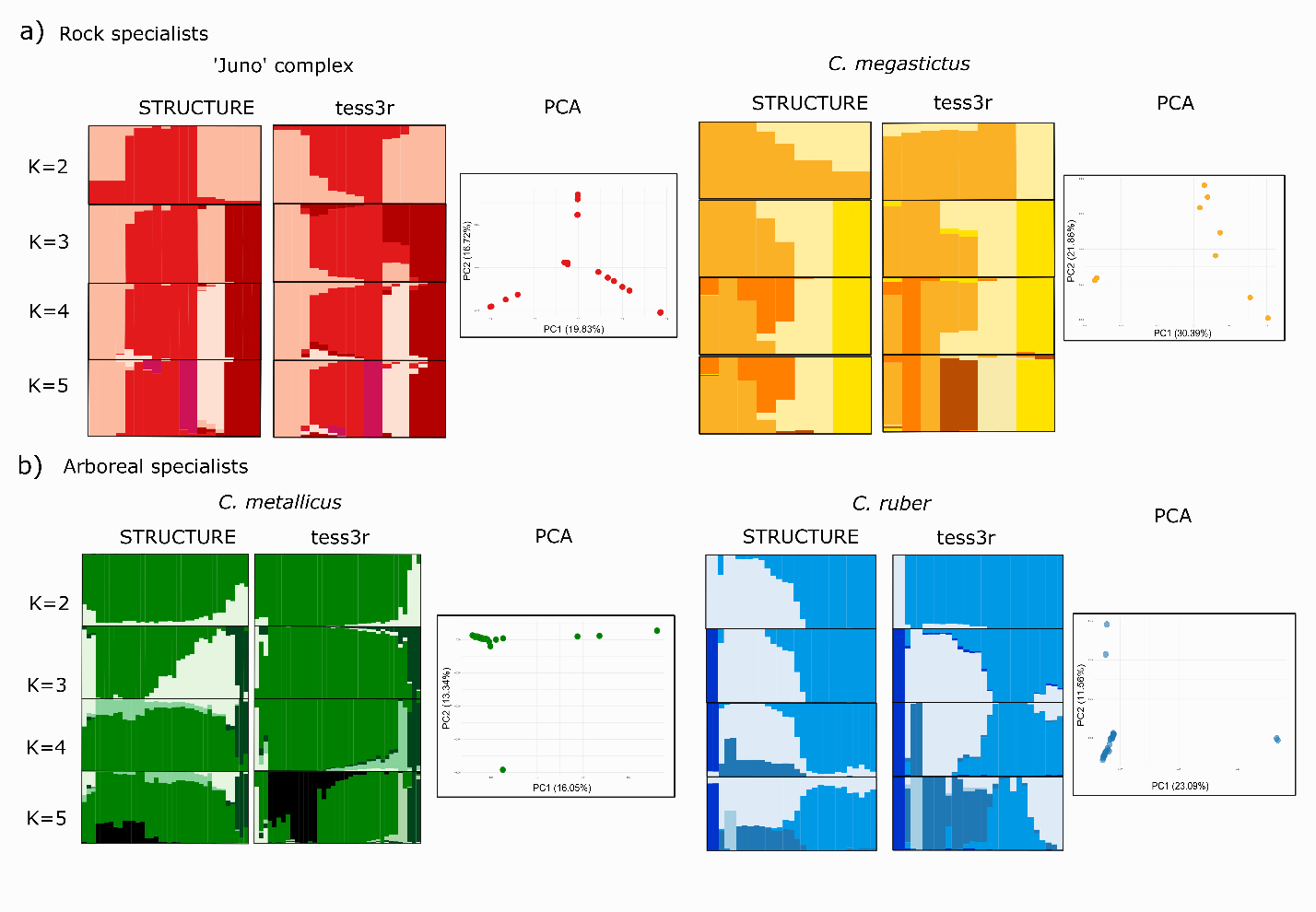


Figure S12. Intraspecific population structure analysis of the focal *Cryptoblepharus* species using the relaxed (all SNPs included) datasets. In a) results of STRUCTURE, Tess3r and PCA analyses for K values ranging from two to five for the rock specialists ‘Juno’ complex and *C. megastictus*. In b), same analyses are shown but for the arboreal species *C. metallicus* and *C. ruber*.


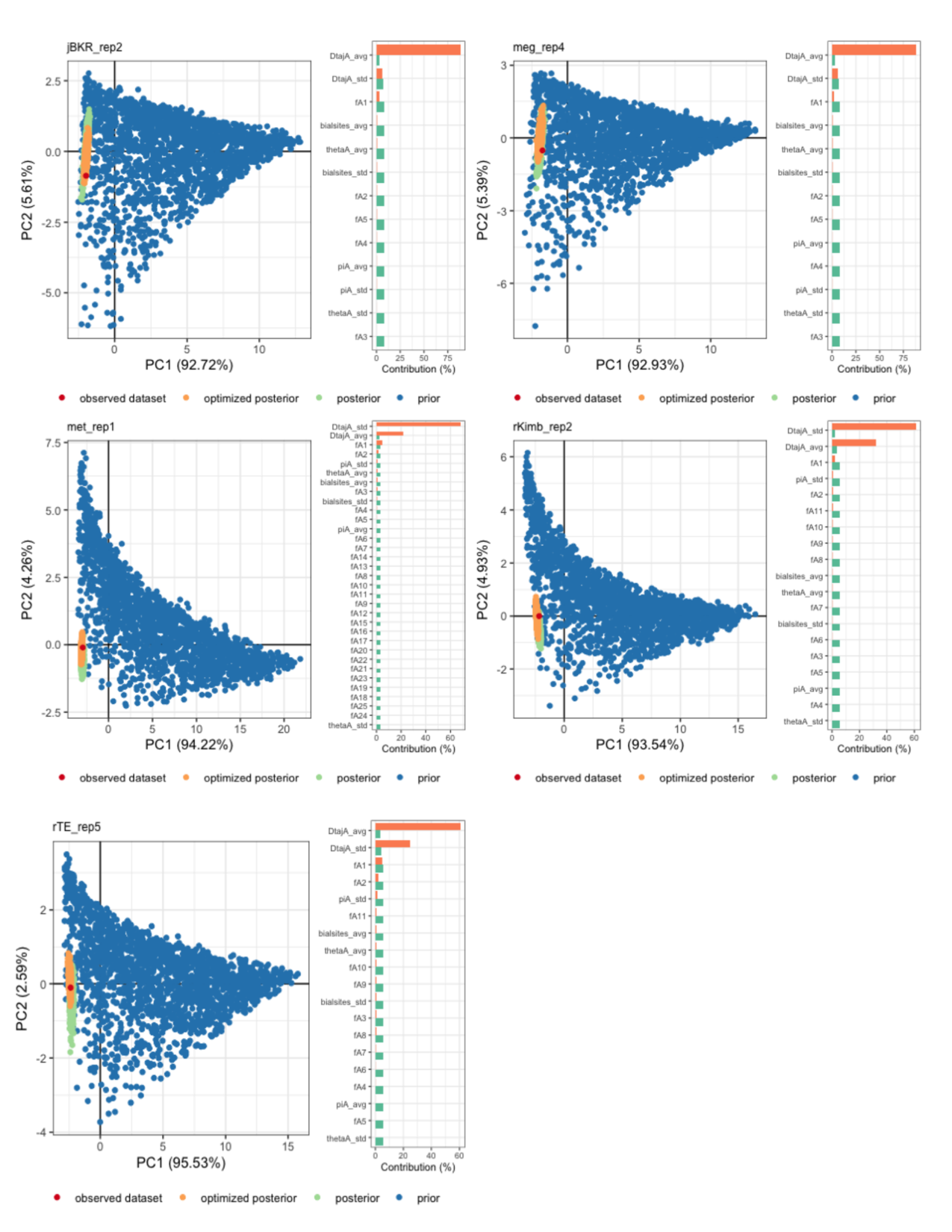


Figure S13. Goodness of fit of single-population models generated by DILS. PCA plots with prior distribution, posterior, optimized posterior and observed dataset are shown for each single-population model. Barplot with PCA contribution (in percentage) of each summary statistic is shown at the right side of each PCA plot (PC1 in orange, PC2 in light green).


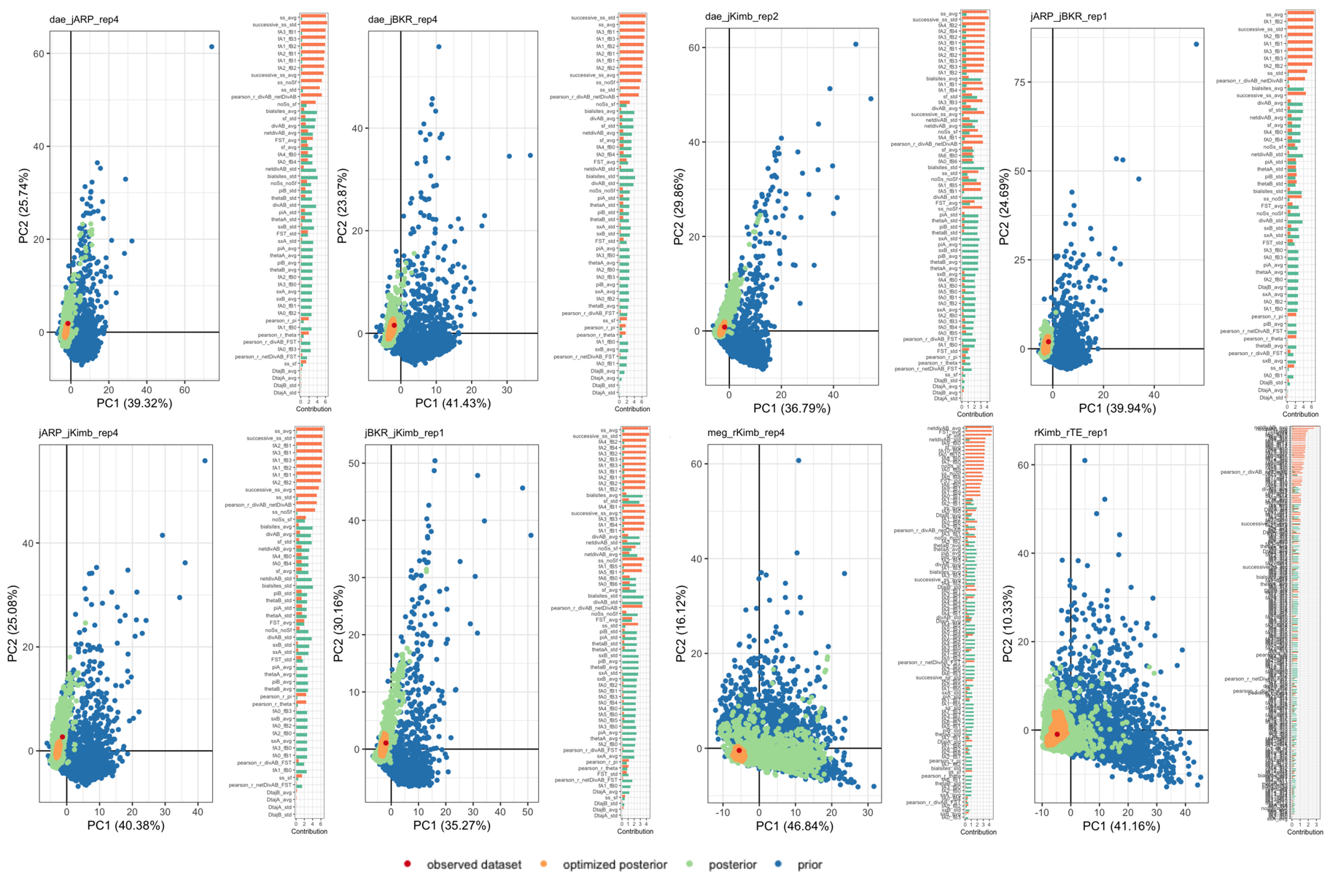
Figure S14. Goodness of fit of two-population models generated by DILS. PCA plots with prior distribution, posterior, optimized posterior and observed dataset are shown for each lineage comparison. Barplot with PCA contribution (in percentage) of each summary statistic is shown at the right side of each PCA plot (PC1 in orange, PC2 in light green).


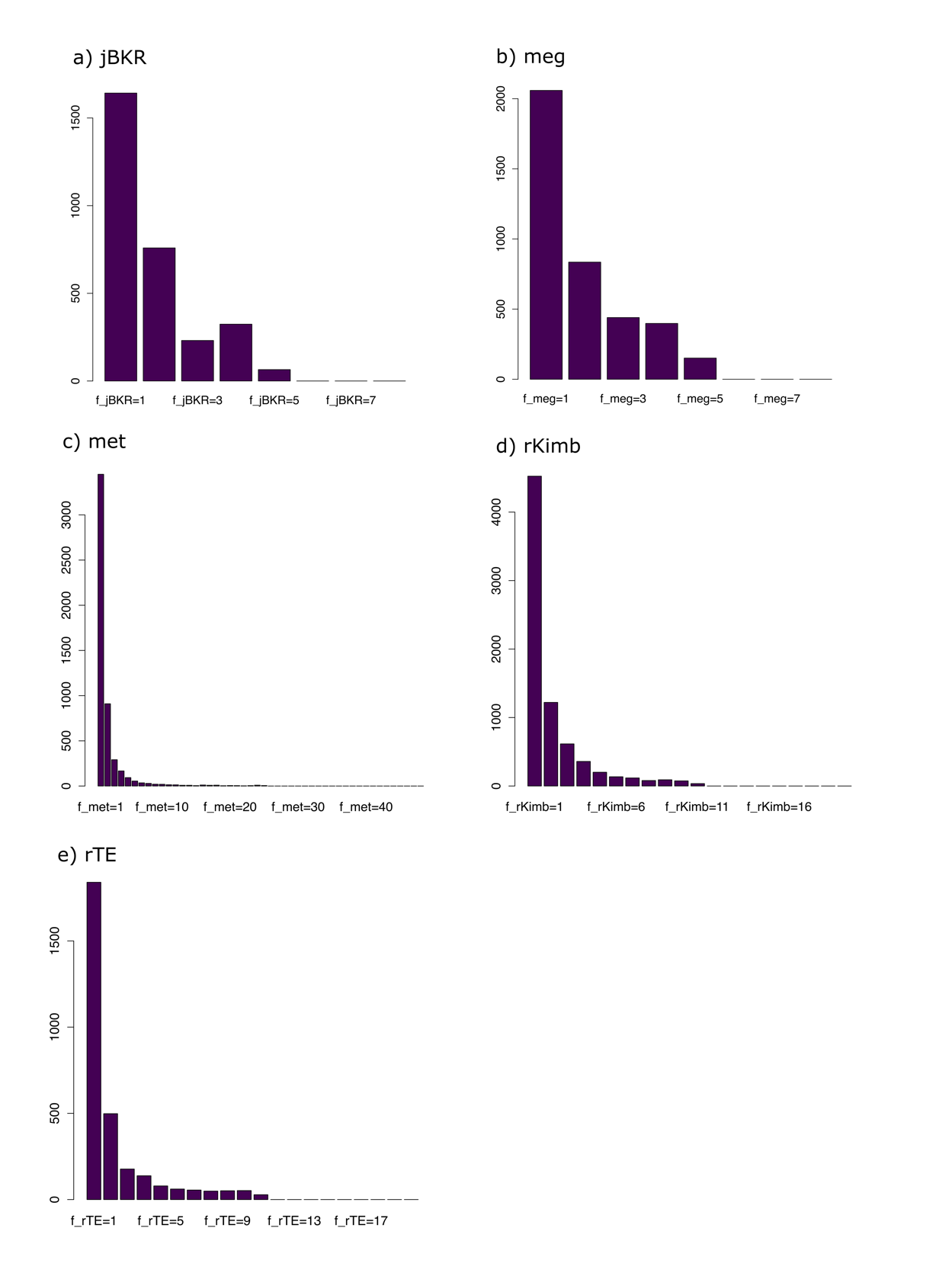


Figure S15. Observed single-population site-frequency-spectrum (SFS). These SFS plots represent one of the summary statistics utilized by DILS to simulate and evaluate the best demographic scenario.


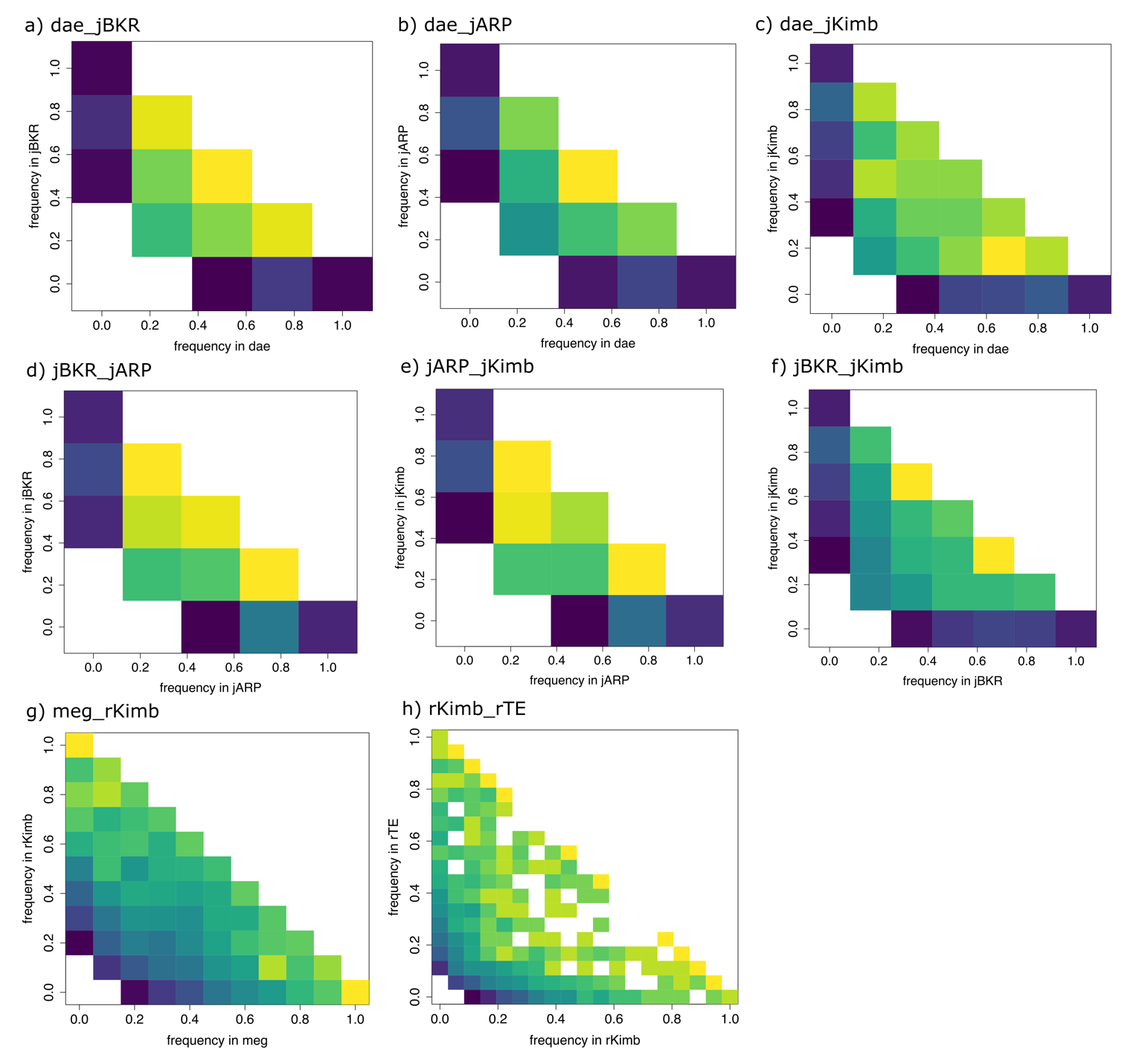
Figure S16. Observed two-population site-frequency-spectrum (SFS). These SFS plots represent one of the summary statistics utilized by DILS to simulate and evaluate the best demographic scenario. Darker colors indicate higher allele frequencies, green shows intermediate allele frequencies and yellow bins point to low allele frequencies.

Table S1. Sample and genetic library information. The table includes museum IDs, tissue and/or field numbers for each sample, collection details (such as location, coordinates and collection year), as well as information about genetic data (e.g. mitochondrial ND2 and nuclear library ID, skink capture version employed, mean individual coverage, total sequence length in bp., number of loci recovered, etc.).

Table S2. Population information and statistics. The table displays the number of individuals included in each population, and the ecotype in which each population is found. Moreover, the table displays the methods used to examine population structure (average individual heterozygosity (H_o_), average nucleotide diversity (π), average genetic diversity (θ) and IBD slope value for each population).

| **Species** | **Population** | **# of individuals** | **Ecotype** | **Average lineage H_O_** | **Average nucleotide diversity within population (π)** | **Watterson's Theta (θ)** | **IBD slope R^2^ (p-value)** |
| --- | --- | --- | --- | --- | --- | --- | --- |
|  |  |  |  |  |  |  |  |
| *C. daedalos* | Daedalos (Dae) | 4 | rock | 0.0024 | 0.0004 | 0.0004 | - |
| *C. juno* | Bullo-Keep River (jBKR) | 8 | rock | 0.0035 | 0.0004 | 0.0004 | 0.298 (0.023) |
|  | Artesian Range-Purnululu (jARP) | 3 | rock | 0.0023 | 0.0003 | 0.0003 | - |
|  | Kimberley (jKimb) | 4 | rock | 0.0020 | 0.0003 | 0.0003 | - |
| *C. megastictus* | Megastictus (Meg) | 9 | rock | 0.0037 | 0.0005 | 0.0006 | 0.301 (0.006) |
| *C. metallicus* | Metallicus (Met) | 37 | arboreal | 0.0038 | 0.0005 | 0.0011 | 0.671 (0.001) |
| *C. ruber* | Top End (TE) | 14 | arboreal | 0.0037 | 0.0004 | 0.0005 | 0.159 (0.003) |
|  | Kimberley (rKimb) | 13 | arboreal | 0.0054 | 0.0006 | 0.0008 | 0.00048 (0.365) |

Table S3. Summary statistics related to population differentiation (Dxy) and fixation (Fst). The table displays values for Fst (upper triangle) and Dxy (lower triangle) between populations.

|  |  | *C. ruber* | | *C. juno / daedalos* | | | | *C. megastictus* | *C. metallicus* |
| --- | --- | --- | --- | --- | --- | --- | --- | --- | --- |
|  |  | rKimb | rTE | dae | jARP | jBKR | jKimb | meg | met |
| *C. ruber* | rKimb |  | 0.20 | 0.63 | 0.65 | 0.64 | 0.66 | 0.11 | 0.46 |
|  | rTE | 0.0006 |  | 0.69 | 0.71 | 0.70 | 0.72 | 0.28 | 0.54 |
| *C. juno / daedalos* | dae | 0.0012 | 0.0012 |  | 0.50 | 0.49 | 0.52 | 0.65 | 0.64 |
|  | jARP | 0.0012 | 0.0012 | 0.0007 |  | 0.47 | 0.47 | 0.67 | 0.64 |
|  | jBKR | 0.0012 | 0.0012 | 0.0007 | 0.0006 |  | 0.51 | 0.66 | 0.64 |
|  | jKimb | 0.0012 | 0.0012 | 0.0007 | 0.0006 | 0.0007 |  | 0.67 | 0.66 |
| *C. megastictus* | meg | 0.0006 | 0.0006 | 0.0013 | 0.0012 | 0.0013 | 0.0013 |  | 0.49 |
| *C. metallicus* | met | 0.001 | 0.0009 | 0.0012 | 0.0012 | 0.0012 | 0.0012 | 0.001 |  |

Table S4. Demographic history. The table displays the name of the populations, the number of individuals used for the demographic analyses, the ecotype in which each population is found, Tajima’s D values and the best supported model in DILS single-population analyses. Stars (*) highlight significant Tajima’s D values and/or demographic scenarios in which the posterior support was greater than 0.85.

| **Species** | **Population** | **Ecotype** | **Tajima’s D** | **DILS 1-pop** |
| --- | --- | --- | --- | --- |
|  |  |  |  |  |
| *C. daedalos* | Daedalos (Dae) | rock | - | - |
| *C. juno* | Bullo-Keep River (jBKR) | rock | -0.519 | Expansion* |
|  | Artesian Range-Purnululu (jARP) | rock | - | - |
|  | Kimberley (jKimb) | rock | - | - |
| *C. megastictus* | Megastictus (Meg) | rock | -0.782 | Expansion* |
| *C. metallicus* | Metallicus (Met) | arboreal | -2.232* | Expansion* |
| *C. ruber* | Top End (rTE) | arboreal | -1.527* | Expansion* |
|  | Kimberley (rKimb) | arboreal | -1.797* | Expansion* |

Table S5. Estimation of Tajima’s D across sites subset. The table displays the name of the populations, the distinct subset of sites used for estimation of Tajima’s D: all sites included, only synonymous sites included and only non-synonymous sites included, and the corresponding values. Moreover, the number of segregating sites included in each estimation are included.

|  | Tajima_D | | |  |
| --- | --- | --- | --- | --- |
|  | all_sites | syn | non_syn |  |
| rKimb | -1.797* | -1.789* | -1.839* |  |
| rTE | -1.527* | -1.597* | -1.560* |  |
| jBKR | -0.519 | -0.524 | -0.754 |  |
| meg | -0.782 | -0.713 | -0.917 |  |
| met | -2.232* | -2.260* | -2.228* |  |
|  | N_seg_sites | | |  |
| rKimb | 4257 | 539 | 3714 |  |
| rTE | 2686 | 354 | 2328 |  |
| jBKR | 1156 | 166 | 989 |  |
| meg | 2076 | 264 | 1809 |  |
| met | 6924 | 987 | 5933 |  |

Table S6. Test of ‘Nmin’ filter in DILS single population analyses. Table shows the number of loci, number of synonymous segregating sites, mean and median locus length as well as total alignment length (including ‘Ns’) across distinct values of minimum number of haplotypes retained per locus (Nmin). Best model and supporting posterior probability are also shown for the best replicate. DILS simulates and evaluates population change under three scenarios: Expansion, Contraction and Expansion.

| Pop | Nmin | Set number | Nloci | nSynSegSite | Mean locus length | Median locus length | Alignment length (including N) | Best rep | Best model | Posterior probability |
| --- | --- | --- | --- | --- | --- | --- | --- | --- | --- | --- |
| jBKR | 2 | set_1 | 486 | 1168 | 1143 | 1017 | 555294 | rep_1 | constant | 0.57 |
| jBKR | 4 | set_7 | 451 | 1977 | 1162 | 1041 | 523863 | rep_3 | expansion | 0.99 |
| jBKR | 6 | set_8 | 438 | 2538 | 1170 | 1041 | 512442 | rep_1 | expansion | 1 |
| jBKR | 8 | set_9 | 427 | 2888 | 1178 | 1065 | 502935 | rep_2 | expansion | 1 |
| jBKR | 10 | set_10 | 398 | 3021 | 1178 | 1059 | 468702 | rep_2 | expansion | 1 |
| meg | 2 | set_1 | 470 | 1798 | 1158 | 1029 | 535017 | rep_4 | expansion | 1 |
| meg | 4 | set_6 | 411 | 2738 | 1195 | 1077 | 496212 | rep_4 | expansion | 0.99 |
| meg | 6 | set_5 | 396 | 3437 | 1207 | 1089 | 477969 | rep_1 | expansion | 1 |
| meg | 8 | set_7 | 369 | 3673 | 1228 | 1125 | 453030 | rep_4 | expansion | 0.99 |
| meg | 10 | set_8 | 347 | 3882 | 1244 | 1125 | 431595 | rep_4 | expansion | 1 |
| meg | 12 | set_9 | 285 | 3539 | 1206 | 1113 | 343593 | rep_5 | expansion | 1 |
| meg | 14 | set_10 | 107 | 1471 | 1185 | 1089 | 126753 | rep_5 | expansion | 1 |
| met | 2 | set_1 | 464 | 1735 | 1166 | 1041 | 535377 | rep_3 | expansion | 1 |
| met | 10 | set_4 | 325 | 3866 | 1269 | 1161 | 412269 | rep_1 | expansion | 1 |
| met | 20 | set_6 | 256 | 4449 | 1327 | 1209 | 339615 | rep_1 | expansion | 1 |
| met | 30 | set_3 | 227 | 4788 | 1369 | 1245 | 296733 | rep_3 | expansion | 1 |
| met | 40 | set_2 | 196 | 4974 | 1408 | 1317 | 275922 | rep_5 | expansion | 0.97 |
| met | 50 | set_5 | 185 | 5175 | 1417 | 1317 | 262227 | rep_1 | expansion | 0.98 |
| met | 56 | set_7 | 171 | 5163 | 1413 | 1317 | 241650 | rep_4 | expansion | 0.97 |
| met | 60 | set_8 | 163 | 5072 | 1363 | 1281 | 222141 | rep_5 | expansion | 0.98 |
| met | 64 | set_9 | 133 | 4332 | 1318 | 1197 | 175227 | rep_4 | expansion | 0.98 |
| met | 68 | set_10 | 98 | 3401 | 1292 | 1167 | 126567 | rep_1 | expansion | 0.97 |
| rTE | 2 | set_1 | 463 | 1328 | 1160 | 1029 | 536883 | rep_3 | expansion | 1 |
| rTE | 4 | set_3 | 399 | 2030 | 1207 | 1089 | 471957 | rep_2 | expansion | 1 |
| rTE | 6 | set_4 | 367 | 2315 | 1231 | 1125 | 451887 | rep_5 | expansion | 0.99 |
| rTE | 8 | set_5 | 355 | 2500 | 1245 | 1137 | 435036 | rep_5 | expansion | 0.99 |
| rTE | 10 | set_6 | 326 | 2458 | 1273 | 1161 | 413676 | rep_5 | expansion | 1 |
| rTE | 14 | set_7 | 316 | 2877 | 1276 | 1161 | 403311 | rep_4 | expansion | 1 |
| rTE | 18 | set_8 | 287 | 2939 | 1301 | 1182 | 373422 | rep_3 | expansion | 0.99 |
| rTE | 22 | set_9 | 267 | 3049 | 1279 | 1173 | 341490 | rep_5 | expansion | 0.99 |
| rTE | 26 | set_10 | 180 | 2303 | 1191 | 1089 | 214341 | rep_4 | expansion | 0.99 |
| rKimb | 2 | set_1 | 490 | 2140 | 1149 | 1029 | 562905 | rep_3 | expansion | 1 |
| rKimb | 4 | set_3 | 455 | 3689 | 1166 | 1041 | 533301 | rep_3 | expansion | 1 |
| rKimb | 6 | set_4 | 429 | 4518 | 1181 | 1053 | 502260 | rep_5 | expansion | 1 |
| rKimb | 8 | set_5 | 412 | 5295 | 1198 | 1077 | 493401 | rep_1 | expansion | 1 |
| rKimb | 10 | set_6 | 399 | 5780 | 1205 | 1077 | 480642 | rep_1 | expansion | 1 |
| rKimb | 14 | set_7 | 381 | 6481 | 1219 | 1101 | 464373 | rep_5 | expansion | 1 |
| rKimb | 18 | set_8 | 356 | 7043 | 1239 | 1125 | 441123 | rep_1 | expansion | 0.99 |
| rKimb | 22 | set_9 | 344 | 7442 | 1240 | 1125 | 426639 | rep_2 | expansion | 1 |
| rKimb | 26 | set_10 | 264 | 6050 | 1211 | 1113 | 319827 | rep_3 | expansion | 0.99 |

Table S7. Test of ‘Nmin’ filter in DILS two population analyses. Table shows the number of loci, number of synonymous segregating sites, mean and median locus length as well as total alignment length (including ‘Ns’) across distinct values of minimum number of haplotypes retained per locus (Nmin). Best model and supporting posterior probability (PP) are also shown for the best replicate. Modes of migration include secondary contact (SC), isolation with migration (IM), ancestral migration (AM) and strict isolation (SI). In DILS hierarchical model selection, ‘Recent migration’ includes SC and IM models, whereas ‘Ancestral migration/Isolation’ includes AM and SI models.

| pop1 | pop2 | Nmin | Set number | Nloci | nSynSegSite | mean locus length | median locus length | alignment length (including N) | Best rep | Best model | PP | Migration Model | PP mig |
| --- | --- | --- | --- | --- | --- | --- | --- | --- | --- | --- | --- | --- | --- |
| dae | jBKR | 2 | set_1 | 447 | 3204 | 1173 | 1041 | 524466 | rep_4 | Recent migration | 0.81 | SC | 0.75 |
| dae | jBKR | 4 | set_2 | 401 | 3856 | 1202 | 1089 | 482124 | rep_4 | Ancestral migration/Isolation | 0.90 | AM | 0.76 |
| dae | jKimb | 2 | set_1 | 453 | 3164 | 1167 | 1041 | 528855 | rep_1 | Recent migration | 0.75 | SC | 0.63 |
| dae | jKimb | 4 | set_3 | 405 | 3718 | 1206 | 1089 | 488595 | rep4 | Ancestral migration/Isolation | 0.87 | AM | 0.81 |
| dae | jKimb | 6 | set_2 | 372 | 3839 | 1208 | 1089 | 449325 | rep_2 | Ancestral migration/Isolation | 0.87 | AM | 0.73 |
| dae | jARP | 2 | set_1 | 447 | 3264 | 1153 | 1029 | 541845 | rep_2 | Recent migration | 0.79 | SC | 0.71 |
| dae | jARP | 4 | set_2 | 437 | 4057 | 1170 | 1041 | 511452 | rep_4 | Ancestral migration/Isolation | 0.86 | AM | 0.75 |
| jBKR | jKimb | 2 | set_1 | 463 | 3207 | 1163 | 1029 | 538290 | rep_2 | Recent migration | 0.80 | IM | 0.52 |
| jBKR | jKimb | 4 | set_3 | 418 | 3909 | 1197 | 1077 | 495681 | rep2 | Ancestral migration/Isolation | 0.93 | AM | 0.62 |
| jBKR | jKimb | 6 | set_2 | 398 | 4320 | 1210 | 1089 | 481752 | rep_1 | Ancestral migration/Isolation | 0.92 | AM | 0.82 |
| jARP | jBKR | 2 | set_1 | 486 | 3292 | 1142 | 1017 | 554883 | rep_5 | Recent migration | 0.66 | SC | 0.71 |
| jARP | jBKR | 4 | set_2 | 439 | 4117 | 1164 | 1041 | 510903 | rep_1 | Ancestral migration/Isolation | 0.89 | AM | 0.77 |
| jARP | jKimb | 2 | set_1 | 479 | 3040 | 1153 | 1029 | 552327 | rep_2 | Recent migration | 0.75 | IM | 0.53 |
| jARP | jKimb | 4 | set_2 | 456 | 3907 | 1164 | 1029 | 529929 | rep_4 | Ancestral migration/Isolation | 0.80 | AM | 0.68 |
| meg | rKimb | 2 | set_1 | 438 | 3665 | 1182 | 1065 | 517857 | rep_3 | Recent migration | 0.57 | IM | 0.52 |
| meg | rKimb | 4 | set_4 | 374 | 4531 | 1216 | 1095 | 454851 | rep_2 | Recent migration | 0.90 | IM | 0.89 |
| meg | rKimb | 6 | set_5 | 336 | 5208 | 1256 | 1143 | 421935 | rep_2 | Recent migration | 0.89 | IM | 0.97 |
| meg | rKimb | 8 | set_6 | 313 | 5644 | 1279 | 1161 | 400179 | rep_1 | Recent migration | 0.91 | IM | 0.97 |
| meg | rKimb | 10 | set_3 | 288 | 5744 | 1306 | 1185 | 376134 | rep_4 | Recent migration | 0.94 | IM | 0.98 |
| meg | rKimb | 12 | set_2 | 229 | 5094 | 1255 | 1149 | 287493 | rep_5 | Recent migration | 0.95 | IM | 0.97 |
| rKimb | rTE | 2 | set_1 | 424 | 3126 | 1191 | 1077 | 505137 | rep_3 | Recent migration | 0.76 | SC | 0.68 |
| rKimb | rTE | 4 | set_8 | 365 | 3828 | 1231 | 1125 | 449472 | rep_1 | Recent migration | 0.91 | IM | 0.91 |
| rKimb | rTE | 6 | set_5 | 321 | 4236 | 1283 | 1161 | 411801 | rep_5 | Recent migration | 0.89 | IM | 0.93 |
| rKimb | rTE | 8 | set_6 | 312 | 4764 | 1282 | 1161 | 399981 | rep_5 | Recent migration | 0.97 | IM | 0.91 |
| rKimb | rTE | 10 | set_4 | 281 | 4791 | 1308 | 1185 | 367494 | rep_5 | Recent migration | 0.98 | IM | 0.91 |
| rKimb | rTE | 14 | set_7 | 247 | 4857 | 1339 | 1233 | 330708 | rep_2 | Recent migration | 0.99 | IM | 0.94 |
| rKimb | rTE | 18 | set_3 | 226 | 5032 | 1373 | 1257 | 310182 | rep_1 | Recent migration | 1 | IM | 0.91 |
| rKimb | rTE | 20 | set_2 | 219 | 5011 | 1346 | 1245 | 294780 | rep_1 | Recent migration | 1 | IM | 0.95 |

Table S8. Summary of results of single-population models indicating support for best replicate and scenario.

| Lineage | Species | Nmin | Best replicate | Best model | Posterior Probability |
| --- | --- | --- | --- | --- | --- |
| junoBKR | *C. juno* | 10 | rep_2 | Expansion | 1.00 |
| megastictus | *C. megastictus* | 10 | rep_4 | Expansion | 1.00 |
| metallicus | *C. metallicus* | 50 | rep_1 | Expansion | 0.98 |
| ruberKimb | *C. ruber* | 22 | rep_2 | Expansion | 1.00 |
| ruberTE | *C. ruber* | 22 | rep_5 | Expansion | 0.99 |

Table S9. Summary of results of two-population models indicating support for best scenario.

| Pop1 | Pop2 | Analysis Type | Nmin | Best replicate | Setp 1: Recent migration versus Ancestral Migration/Isolation: | Step 1: best model posterior probability | Step 2: Migration model | Step 2: Migration model posterior probability |
| --- | --- | --- | --- | --- | --- | --- | --- | --- |
| dae | jARP | Between species | 4 | rep_4 | Ancestral migration/Isolation | 0.86 | AM | 0.75 |
| dae | jBKR | Between species | 4 | rep_4 | Ancestral migration/Isolation | 0.9 | AM | 0.76 |
| dae | jKimb | Between species | 6 | rep_2 | Ancestral migration/Isolation | 0.87 | AM | 0.73 |
| jARP | jBKR | Within species | 4 | rep_1 | Ancestral migration/Isolation | 0.89 | AM | 0.77 |
| jARP | jKimb | Within species | 4 | rep_4 | Ancestral migration/Isolation | 0.8 | AM | 0.68 |
| jBKR | jKimb | Within species | 6 | rep_1 | Ancestral migration/Isolation | 0.92 | AM | 0.82 |
| meg | rKimb | Between species | 10 | rep_4 | Recent migration | 0.94 | IM | 0.98 |
| rKimb | rTE | Within species | 18 | rep_1 | Recent migration | 1.00 | IM | 0.91 |

Table S10. Table with a subset of the summary statistics from the single-population DILS ABC models. For a more detailed summary of the statistics used for the analyses, please refer to the Dryad.

| analysis | rep | piA_avg | thetaA_avg | DtajA_avg | bialsites_avg |
| --- | --- | --- | --- | --- | --- |
| jBKR_rep2 | rep2 | 0.00982 | 0.01148 | -0.61243 | 7.59045 |
| meg_rep4 | rep4 | 0.01518 | 0.01722 | -0.53433 | 11.25507 |
| met_rep1 | rep1 | 0.00953 | 0.02954 | -2.21262 | 27.97297 |
| rKimb_rep2 | rep2 | 0.01559 | 0.0262 | -1.51441 | 21.73469 |
| rTE_rep5 | rep5 | 0.00913 | 0.01479 | -1.30172 | 11.42642 |

| analysis | rep | piA_avg | piB_avg | thetaA_avg | thetaB_avg | netdivAB_avg | divAB_avg | FST_avg | DtajA_avg | DtajB_avg | pearson_r_pi | pearson_r_theta | pearson_r_divAB_netDivAB | pearson_r_divAB_FST | pearson_r_netDivAB_FST |
| --- | --- | --- | --- | --- | --- | --- | --- | --- | --- | --- | --- | --- | --- | --- | --- |
| dae_jARP | rep4 | 0.0088 | 0.00872 | 0.00879 | 0.00853 | 0.01052 | 0.01928 | 0.38567 | 0.03387 | 0.19642 | 0.29688 | 0.33609 | 0.87504 | 0.32344 | 0.64096 |
| dae_jBKR | rep4 | 0.00885 | 0.00965 | 0.00896 | 0.00983 | 0.01077 | 0.02002 | 0.38198 | -0.06482 | -0.1488 | 0.18642 | 0.19884 | 0.87029 | 0.32553 | 0.65559 |
| dae_jKimb | rep2 | 0.00845 | 0.0085 | 0.00868 | 0.00812 | 0.01027 | 0.01874 | 0.37305 | -0.10966 | 0.20955 | 0.32578 | 0.37644 | 0.88345 | 0.42091 | 0.73037 |
| jARP_jBKR | rep1 | 0.00869 | 0.00989 | 0.00857 | 0.01017 | 0.00937 | 0.01866 | 0.34829 | 0.16047 | -0.22502 | 0.19926 | 0.19469 | 0.85126 | 0.32029 | 0.69013 |
| jARP_jKimb | rep4 | 0.00864 | 0.00856 | 0.00849 | 0.00848 | 0.00847 | 0.01707 | 0.34588 | 0.18493 | 0.12065 | 0.44334 | 0.43795 | 0.82362 | 0.29699 | 0.68796 |
| jBKR_jKimb | rep1 | 0.00967 | 0.0084 | 0.01049 | 0.00806 | 0.00988 | 0.01892 | 0.35642 | -0.39034 | 0.20552 | 0.21395 | 0.22358 | 0.87824 | 0.35703 | 0.68731 |
| meg_rKimb | rep4 | 0.01363 | 0.01599 | 0.01561 | 0.02122 | 0.0019 | 0.01671 | 0.0654 | -0.56353 | -1.11417 | 0.66265 | 0.65136 | 0.41838 | 0.04596 | 0.82054 |
| rKimb_rTE | rep1 | 0.01378 | 0.00773 | 0.02271 | 0.01194 | 0.00189 | 0.01264 | 0.07641 | -1.46748 | -1.2058 | 0.57263 | 0.53908 | 0.6209 | 0.27751 | 0.81322 |

Table S11. Table with a subset of the summary statistics from the two-population DILS ABC models. For a more detailed summary of the statistics used for the analyses, please refer to the Dryad.
